# Low Doses of Imatinib Induce Myelopoiesis and Enhance Host Anti-microbial Immunity

**DOI:** 10.1371/journal.ppat.1004770

**Published:** 2015-03-30

**Authors:** Ruth J. Napier, Brian A. Norris, Alyson Swimm, Cynthia R. Giver, Wayne A. C. Harris, Julie Laval, Brooke A. Napier, Gopi Patel, Ryan Crump, Zhenghong Peng, William Bornmann, Bali Pulendran, R. Mark Buller, David S. Weiss, Rabindra Tirouvanziam, Edmund K. Waller, Daniel Kalman

**Affiliations:** 1 Microbiology and Molecular Genetics Graduate Program, Emory University School of Medicine, Atlanta, Georgia, United States of America; 2 Immunology and Molecular Pathogenesis Graduate Program, Emory University School of Medicine, Atlanta, Georgia, United States of America; 3 Department of Pathology and Laboratory Medicine, Emory University School of Medicine, Atlanta, Georgia, United States of America; 4 Department of Hematology and Medical Oncology, Winship Cancer Institute, Emory University, Atlanta, Georgia, United States of America; 5 Department of Pediatrics, Emory University School of Medicine, Atlanta, Georgia, United States of America; 6 Center for Cystic Fibrosis Research, Children’s Healthcare of Atlanta, Atlanta, Georgia, United States of America; 7 Institut de Génétique Moléculaire de Montpellier (IGMM), CNRS UMR5535, Université Montpellier, Montpellier, France; 8 Department of Molecular Microbiology and Immunology, Saint Louis University, St. Louis, Missouri, United States of America; 9 MD Anderson Cancer Center, University of Texas, Houston, Texas, United States of America; 10 Emory Vaccine Center, Emory University, Atlanta, Georgia, United States of America; 11 Yerkes National Primate Research Center, Atlanta, Georgia, United States of America; 12 Division of Infectious Diseases, Department of Medicine, Emory University School of Medicine, Atlanta, Georgia, United States of America; University of Massachusetts, UNITED STATES

## Abstract

Imatinib mesylate (Gleevec) inhibits Abl1, c-Kit, and related protein tyrosine kinases (PTKs) and serves as a therapeutic for chronic myelogenous leukemia and gastrointestinal stromal tumors. Imatinib also has efficacy against various pathogens, including pathogenic mycobacteria, where it decreases bacterial load in mice, albeit at doses below those used for treating cancer. We report that imatinib at such low doses unexpectedly induces differentiation of hematopoietic stem cells and progenitors in the bone marrow, augments myelopoiesis but not lymphopoiesis, and increases numbers of myeloid cells in blood and spleen. Whereas progenitor differentiation relies on partial inhibition of c-Kit by imatinib, lineage commitment depends upon inhibition of other PTKs. Thus, imatinib mimics “emergency hematopoiesis,” a physiological innate immune response to infection. Increasing neutrophil numbers by adoptive transfer sufficed to reduce mycobacterial load, and imatinib reduced bacterial load of *Franciscella spp.*, which do not utilize imatinib-sensitive PTKs for pathogenesis. Thus, potentiation of the immune response by imatinib at low doses may facilitate clearance of diverse microbial pathogens.

## Author Summary

Host-directed therapeutics (HDTs) for infectious diseases target cellular mechanisms used by pathogens to move into, through, or out of cells. The Abl tyrosine kinase (TK) inhibitor and cancer therapeutic imatinib mesylate (Gleevec), for example, has activity against bacterial and viral pathogens via effects on pathogen entry (polyomaviruses), intracellular transit (*Mycobacteria*) and exit (poxviruses and filoviruses). Other HDTs target the host immune system by suppressing or activating circulating innate and adaptive cells. Here we report that imatinib at doses that are effective in clearing Mycobacterial infections but which are 10-fold lower than those used for cancer, mimics a physiological innate response to infection in the bone marrow, called the “emergency response,” in which hematopoietic stem cells and multipotent progenitors expand and differentiate into mature myeloid cells that migrate to peripheral sites. Imatinib effects occur in part via partial inhibition of c-Kit, suggesting a mechanism by which c-Kit controls the earliest stages of hematopoiesis. Mimicking a physiological antimicrobial response may make imatinib broadly useful. Accordingly, imatinib also has efficacy against infections caused by *Franciscella spp*., which do not use imatinib-sensitive TKs for pathogenesis. These observations identify myelopoiesis as an important target for HDTs, and provide information on how to dose imatinib for clinical use.

## Introduction

Signaling by protein tyrosine kinases (PTKs) mediates a variety of cellular processes including migration, morphogenesis, stress responses, and cytoskeletal reorganization [1,2]. Dysregulation of PTK activity causes a variety of diseases, including cancer. One such cancer, chronic myelogenous leukemia (CML), is associated with a characteristic translocation between chromosomes 9 and 22, called the “Philadelphia chromosome (Ph),” which encodes a fusion protein composed of breakpoint cluster region (BCR) protein and the PTK Abl1, called BCR-ABL[3]. Expression of BCR-ABL in hematopoietic stem cells (HSCs) results in aberrant proliferation of Ph^+^ stem cells and the accumulation of myeloid cells in the bone marrow and blood.

Over the last decade, development of small molecule inhibitors of Abl1 and BCR-ABL, such as imatinib mesylate (imatinib, Gleevec), have dramatically reduced mortality rates in patients with CML and related cancers [4–8]. Imatinib selectively kills Ph^+^ myeloid lineage cells in the bone marrow and periphery, whose survival depends on expression of BCR-ABL. However, the drug does not appear to affect survival of Ph^+^ hematopoietic stem cells (HSCs), nor of Ph^-^ cells [9].

Imatinib inhibits several other structurally related PTKs in a dose-dependent manner [10–13]. Nanomolar concentrations of imatinib inhibit c-Abl1 and c-Abl2, platelet-derived growth factor receptor alpha (PDGFRα) and beta (PDGFRβ), and the stem cell receptor (c-Kit), whereas micromolar concentrations inhibit macrophage colony-stimulating factor receptor (m-CSFR or c-fms)[13]. Accordingly, imatinib also has efficacy against gastrointestinal stromal tumors (GISTs), which are caused by dysregulated c-Kit or PDGFRα [14].

Various pathogens utilize activation of Abl1 and related PTKs to facilitate intracellular survival, intracellular trafficking, and spread from cell to cell [15]. These include diarrheagenic *Escherichia coli*, *Pseudomonas*, *Salmonella*, *Shigella*, *Helicobacter*, *Anaplasma*, *Chlamydia*, and pathogenic mycobacteria amongst bacteria, and filoviruses, HIV, Coxsackie virus, Kaposi sarcoma virus, Polyomaviruses, and orthopoxviruses amongst viruses, as well as the human parasite *Leishmania* [16–33]. For pathogenic mycobacteria including *Mycobacterium tuberculosis* (Mtb) and *Mycobacterium marinum* (Mm), imatinib enhances trafficking of the bacteria into acidified vesicles [30,33], whereas for orthopoxviruses, the drug prevents Abl-dependent dissemination of the virus [31,32].

In contrast to the wealth of information on the role of PTKs in cancer and microbial pathogenesis, information on how PTK inhibitors function *in vivo* remains more limited. Historically, the therapeutic effects of imatinib have been attributed to its cell autonomous effects on tumor cells expressing oncogenic kinases, or to its inhibition of cellular kinases and pathogenesis in infected cells. However, recent evidence suggests that imatinib also regulates the immune response. Imatinib inhibits T cell signaling *in vitro*, and reportedly causes immunosuppression and even neutropenia in some patients, especially at high doses [34]. By contrast, other data suggests that the therapeutic effect of imatinib may actually require the immune system. Thus, imatinib remains effective *in vivo* even against engrafted GIST cells that are unresponsive to the drug *in vitro*, an effect attributed to stimulation of cross talk between dendritic cells and natural killer cells, which have attendant anti-tumor activity [35]. Moreover, recent reports indicate that imatinib relieves c-Kit-dependent immunosuppression by regulatory T cells, which in turn potentiate anti-tumor CD8^+^ T cell responses [36].

Such immunostimulatory effects may be important for infectious diseases as well. We measured the effects of imatinib on innate immune responses, particularly the increased numbers of myeloid cells typically seen following bacterial infection. We demonstrate that low doses of imatinib activate myelopoiesis in the bone marrow and increase the number of myeloid cells in the bone marrow, blood, and spleen, which enhances a physiological antimicrobial response to infection.

## Results

### Imatinib induces expansion of myeloid cells in spleen and blood

**Fig 1:**
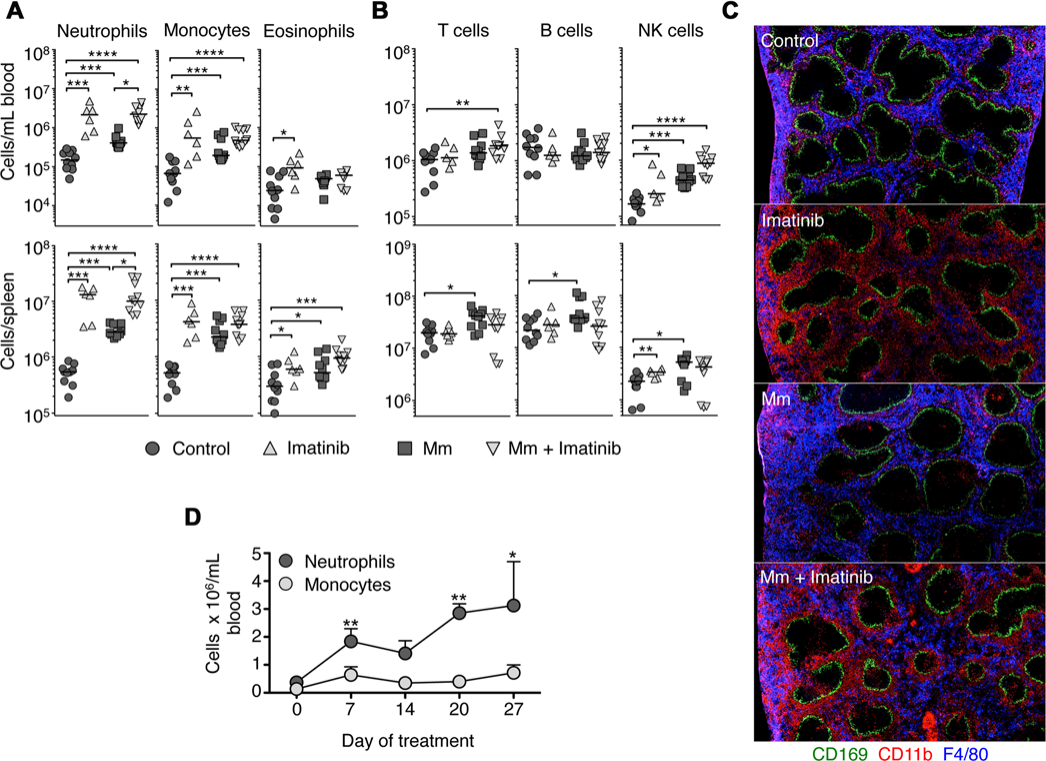
Imatinib treatment induces expansion of myeloid cells in mouse spleen and blood. C57Bl/6 mice were administered imatinib at 66mg/kg/day (Imatinib) or left untreated (Control). For some experiments, mice were either injected in the tail vein with 10^5^ CFU Mm 1218R one day after administration of drug (Mm + imatinib) or carrier (Mm). Neutrophils (CD11b^+^ Ly6C^int^ Gr-1^hi^ SSC^int^), monocytes (CD11b^+^ Ly6C^hi^ Gr-1^int^ SSC^low^ and F4/80^+^) and eosinophil (CD11b^+^ Ly6C^lo^ Gr-1^int^ SSC^hi^ and F4/80^+^) **(A),** or T cells (Thy1.2^+^), B cells (B220^+^ CD19^+^) and NK cells (NK1.1^+^) **(B)** were enumerated by flow cytometry in the blood (top panel) or spleen (bottom panel) at d7 post infection or treatment. Each symbol represents one mouse. Data shown are representative of six independent experiments. **(C)** Sections of spleens taken from mice left untreated or treated with imatinib at 66mg/kg/day plus or minus infection with Mm. Sections were stained with anti-CD169 to recognize marginal zone macrophages (green), anti-F4/80 to recognize red pulp myeloid cells (blue), and anti-CD11b to recognize neutrophils and monocytes (red). **(D)** C57Bl/6 mice were administered imatinib at 66 mg/kg/day for 28 days. On day 0, 7, 14, 21, 28 of treatment, mice were bled from the tail vein and numbers of neutrophils and monocytes enumerated by flow cytometry (three mice per time point). Data shown are from two independent experiments. For all data sets, the line in each group of data points represents the median. For **A**, and **B**, a Mann-Whitney test was used for pairwise comparisons (e.g. +/- drug or +/- infection only), and a Kruskal Wallis test for multiple comparisons across groups. For all panels, p values less than 0.05 were considered significant; p values < 0.05 are denoted by *, values <.001 by **, and values <.0001 by ***, and values <.00005 by ****.

Previous studies have shown that imatinib maximally reduces mycobacterial load in mice when administered at 66mg/kg/d, whereas higher doses (e.g. 200mg/kg/d) proved much less effective [30]. Doses of 66mg/kg/d resulted in steady state serum levels of 57+/-21ng/ml (~100nM) in mice. Such serum levels would be considered sub-therapeutic in humans, where doses of 400mg QD result in serum levels of ~1500 to ~3000 ng/ml (2–5 μM)[37,38]. To characterize the effect of imatinib at a dose of 66mg/kg/d on the composition of immune cells, the drug was delivered to uninfected mice or those infected with Mm beginning one day prior to infection and continuing for the duration of the experiment. Blood and spleens were harvested seven days after infection and lymphoid, myeloid and dendritic cell (DC) populations enumerated by flow cytometry (Figs. 1A,B; S1A-S1C). Imatinib significantly increased the number of myeloid-derived cells in the blood and spleen in both infected and uninfected animals. No difference in cell numbers was evident in animals left untreated or treated with the carrier (water). The number of neutrophils, defined as CD11b^+^ Ly6C^int^ Gr-1^hi^ SSC^int^, increased on average by 14-fold in blood, and 22-fold in spleen in imatinib-treated mice compared to uninfected untreated controls (Fig. 1A; representative plots in S1B Fig.). Infection with Mm alone increased neutrophil numbers by ~3 and 6-fold in the blood and spleen, respectively. Neutrophil numbers in animals treated with imatinib and infected increased by 15- and 25-fold in blood and spleen, respectively, compared to numbers observed in uninfected controls (Figs. 1A; S1B). Monocytes, eosinophils, natural killer (NK) cells, and CD8^−^ DCs likewise increased in number following imatinib treatment with or without infection, though to a lesser extent than neutrophils (Figs. 1A,B; S1C). Imatinib also increased numbers of CD8^+^DCs in the spleen compared to uninfected controls (S1C Fig). By contrast, imatinib produced no change in the numbers of T cells or B cells (Fig. 1B). Thus, imatinib caused a large-scale increase in cells of the myeloid lineage, as well as changes in CD8^+^ DCs and NK cells.

To validate the expansion of the myeloid compartment, histological sections of spleens were analyzed seven days after imatinib treatment and/or infection with Mm. As shown in Fig. 1C, the splenic architecture was largely maintained with drug treatment or infection, with CD169^+^ marginal zone macrophages (green) clearly demarcating lymphocyte areas from F4/80^+^ CD11b^+^ red pulp (blue, Fig. 1C). However, the expansion of myeloid cells following imatinib treatment was clearly evident, with accumulation of CD11b^+^ cells evident (red, Fig. 1C), particularly in the areas of the red pulp adjacent to the marginal zones.

To determine whether the number of myeloid cells with imatinib transiently increased, uninfected mice were treated with drug for up to 27 days, the longest time tested, and the numbers of immune cells in the blood determined at weekly intervals. Increases in numbers of neutrophils and monocytes persisted for as long as the drug was administered, whereas the numbers of eosinophils, DCs, B cells and T cells did not significantly increase over this time period (e.g. Fig. 1D).

### Imatinib increases number of myeloid cells in the bone marrow

**Fig 2:**
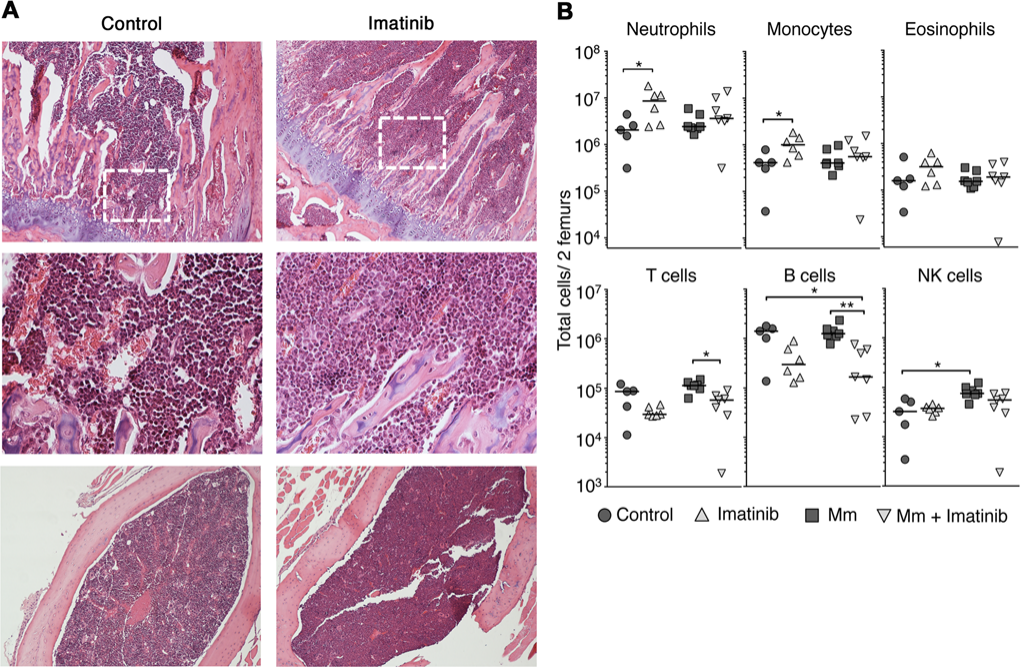
Effects of imatinib on neutrophils and monoctyes in bone marrow. **(A)** Geimsa staining of femurs from C57Bl/6 mice administered imatinib at 66 mg/kg/d for seven days or left untreated as indicated. Top panels: coronal sections (10X). Middle panels: Insets (40x) derived from the area denoted by a dashed white box. Bottom panels: sagittal sections (10x). **(B)** C57Bl/6 mice were administered imatinib at 66 mg/kg/d for seven days or left untreated, as indicated. Beginning 24h after onset of drug, mice were either injected in the tail vein with 10^5^ CFU Mm 1218R or left uninfected. At seven days post-treatment bone marrow was collected from femurs. Data shown are representative of three independent experiments. For all data sets, the line in each group of data points represents the median. A Mann-Whitney test was used for pairwise comparisons, and a Kruskal Wallis test for multiple comparisons.

We next determined whether the increased number of myeloid cells in blood and spleen seen at 66mg/kg/d resulted from increased production in the bone marrow. Giemsa staining of femurs from imatinib-treated mice revealed a pronounced increase in marrow cellularity (Fig. 2A). Moreover, centrifuged pellets of bone marrow from mice treated with imatinib appeared white, a hallmark of neutrophilia, compared to marrow from untreated mice, which was pink. The numbers of neutrophils and monocytes in bone marrow from imatinib-treated mice increased by 4- and 3-fold, respectively (Fig. 2B; gating schema in S2A Fig). The numbers of mature B cells, T cells, DCs, eosinophils, and NK cells in the bone marrow did not change significantly upon treatment with imatinib (Figs. 2B; S2B). Thus, accumulation of mature myeloid cells in the marrow was positively correlated with increases in mature cells in the blood and spleen at a dose of 66mg/kg/d. By contrast, with infection the number of mature myeloid cells in the bone marrow did not significantly increase with or without imatinib. Because increases in numbers of mature cells were evident in blood and peripheral tissues (Fig. 1), we surmise that infection augmented migration of mature cells from the bone marrow.

### Imatinib increases numbers of multipotent progenitors in the bone marrow

The bone marrow is the primary site of post-natal hematopoiesis, where dormant hematopoietic stem cells (HSC) become activated [39,40] and sequentially differentiate into four identifiable multipotent progenitor cell types (MPP1-MPP4)[41], myeloid/lymphoid or erythroid/myeloid progenitors [42,43], and finally, into a variety of both immature cells and mature cells, which migrate out of the bone marrow. HSCs are phenotypically distinguished from more mature cells primarily based on their capacity for efficient immune reconstitution upon transplant into lethally irradiated animals [39,40].

**Fig 3:**
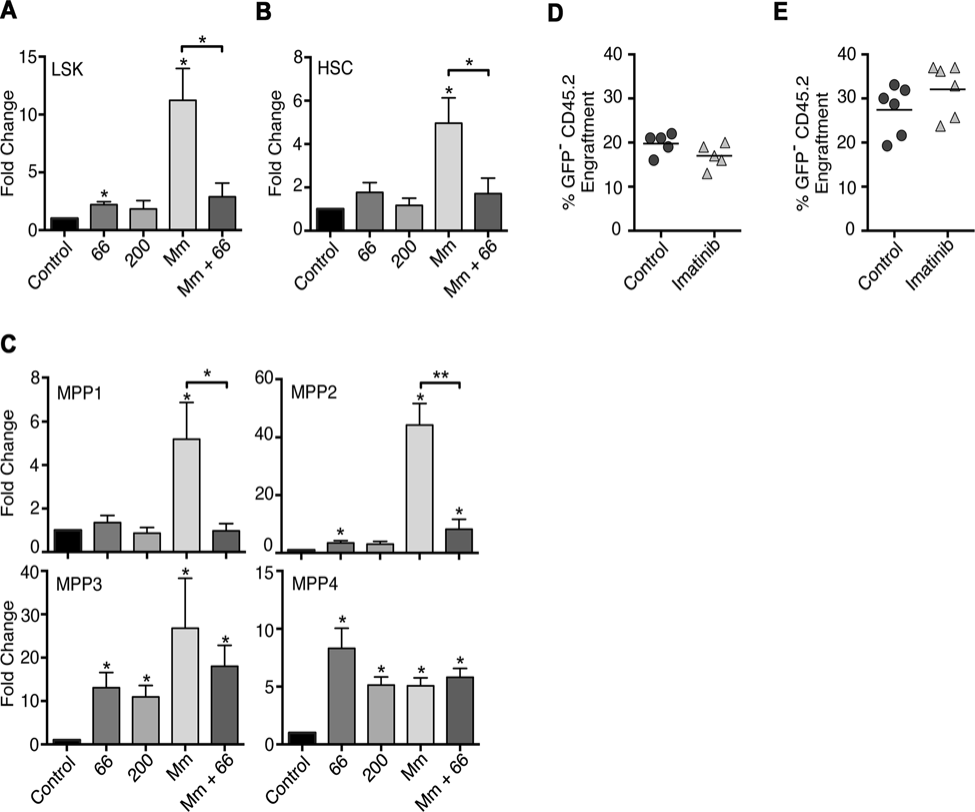
Effects of Imatinib on HSCs and multipotent progenitors *in vivo*. **(A-C)** C57Bl/6 mice were administered imatinib at 66 or 200mg/kg/d or left untreated. Beginning 24h post-treatment mice were either injected in the tail vein with 10^5^ CFU Mm 1218R or left uninfected. Bone marrow was collected from femurs and HSCs and MPPs enumerated by flow cytometry[41]. **(A-C)** Effect of imatinib or infection on Lin^neg^sca1^+^CD117^+^ (LSK) cells (**A**), HSCs (CD34^−^CD48^−^CD150^+^CD135^−^) (**B**) or MPP1 (CD34^+^CD48^-^CD150^+^CD135^−^), MPP2 (CD34^+^CD48^+^CD150^+^CD135^−^), MPP3 (CD34^+^CD48^+^CD150^−^CD135^−^) and MPP4 (CD34^+^CD48^+^CD150^−^CD135^+^) cells (**C**). Each bar represents the average fold change relative to control in 2–6 independent experiments with 3–6 animals per condition per experiment. An asterisk (*) above the error bar indicates statistical significance compared to control as determined by t-test. Statistical significance was also assessed for infected (Mm) versus infected, drug treated animals (Mm + imatinib) and is indicated by a bracket over the bars. **(D-E)** Competitive bone marrow transplants with whole marrow (**D**) or LSK cells (**E**) from control animals or animals treated with 66mg/kg/d imatinib for 7d. **(D)** Congenic CD45.1^+^ C57Bl/6 mice were lethally irradiated and transplanted with 2 x 10^6^ whole bone marrow cells from untreated GFP^+^ C57Bl/6 donors mixed with 2 x 10^6^ whole bone marrow cells from C57Bl/6 (CD45.2) donors that were either treated with imatinib for seven days or left untreated. Numbers of leukocytes derived from the different donor sources or the recipient mice were determined by flow cytometry at 4 months post transplant (n = 5 recipient mice per condition). **(E)** GFP^+^ C57Bl/6 marrow (3x10^5^ cells) was mixed with 5x10^3^ sorted LSK cells before transplantation, and donor chimerism was determined using blood drawn 4 months post-transplantation (n = 5 recipient mice per condition). A Mann-Whitney nonparametric test was used to determine significance.

We next assessed the effects of imatinib on numbers of neutrophil precursors, HSCs, and multipotent progenitors in bone marrow by flow cytometry. Despite increases in the numbers of mature neutrophils in the bone marrow, no effects of imatinib were evident on the number of neutrophil precursors, including promyelocytes, myelocytes, and metamyelocytes (S3A and S3B Fig). By contrast, the fraction of Lineage^neg^Sca1^+^c-Kit^+^ (LSK) cells [44], which include MPPs and HSCs, increased by 2-fold on average with imatinib treatment at 66 mg/kg/day, and to an even greater extent with infection (Figs. 3A; S4A and S4B). Notably accumulation of LSK cells evident with infection markedly decreased with imatinib treatment (Fig. 3A). Using markers defined by Wilson *et al*. [41] (S4A Fig), we next determined whether the observed increase in numbers of LSK cells was due to an effect on HSCs, MPP1, MPP2, MPP3, or MPP4 cells. As shown in Fig. 3B, the number of HSCs remained unchanged with imatinib alone at 66 mg/kg/day, but increased by up to 5-fold with Mm infection. Notably, reduced accumulation of HSCs in infected animals was evident upon treatment with imatinib. Numbers of MPP2, MPP3 and MPP4 cells increased with imatinib at 66 mg/kg/day, with the greatest accumulation compared to control animals evident with MPP3 and MPP4 cells (Fig. 3C). Infection alone produced an accumulation of MPP1, MPP2, MPP3, and MPP4 cells, and, as with HSCs, MPP1 and MPP2 cells accumulated to a lesser extent with infection plus imatinib compared to infection alone (Fig. 3C). Together, these data indicate that (i) imatinib at 66 mg/kg/day does not induce accumulation of HSCs, whereas infection does; (ii) imatinib nevertheless reduces accumulation of HSCs upon infection, suggesting that the drug may increase flux of HSCs to progenitors; and (iii) imatinib regulates accumulation of subsets of MPPs, an effect also evident with infection.

### Imatinib does not increase numbers of transplantable HSCs

To determine whether imatinib caused an expansion of transplantable HSCs, we assessed the engraftment capacity of these cells in a competitive repopulation assay [45]. Marrow derived from control CD45.2 C57Bl/6 mice or CD45.2 C57Bl/6 mice treated with imatinib for seven days was mixed with marrow derived from untreated CD45.2 GFP^+^C57Bl/6 mice. Irradiated congenic CD45.1^+^ C57Bl/6 mice were then injected with either the control/GFP mixture or the imatinib/GFP mixture. At 4 and 12 months post-transplant, mice were bled and the relative numbers of blood cells derived from GFP-, naïve- or imatinib-treated donor mice, or recipient mice determined by flow cytometry. At all time points, the proportion of blood cells derived from naïve or imatinib-treated animals were similar, indicating a lack of competitive advantage of bone marrow from imatinib-treated mice (Fig. 3D), in accordance with the absence of a statistically significant accumulation of HSCs (Fig. 3B). Because HSCs represent less than ~0.003% of the total marrow [41], it remained possible that the competition assay with whole marrow might not resolve small differences in the HSC numbers between imatinib-treated or naive mice. To assess this possibility, competitive transplant experiments using just the LSK fraction from naïve or imatinib-treated animals were performed. As shown in Fig. 3E, at four months post transplant, proportions of mature cells derived from imatinib-treated mice were unchanged compared to their naïve counterparts. Together, these data suggest that imatinib does not cause an increase in the number of transplantable HSCs, in accordance with observations showing that HSCs do not accumulate upon treatment with the drug (Fig. 3B). However, these data do not rule out the possibility that imatinib increases the capacity of a fraction of activated HSCs to asymmetrically divide and rapidly differentiate into MPPs, a result suggested by data with infection plus imatinib (Fig. 3A-C).

### Imatinib increases myeloid progenitors in mouse and human marrow

**Fig 4:**
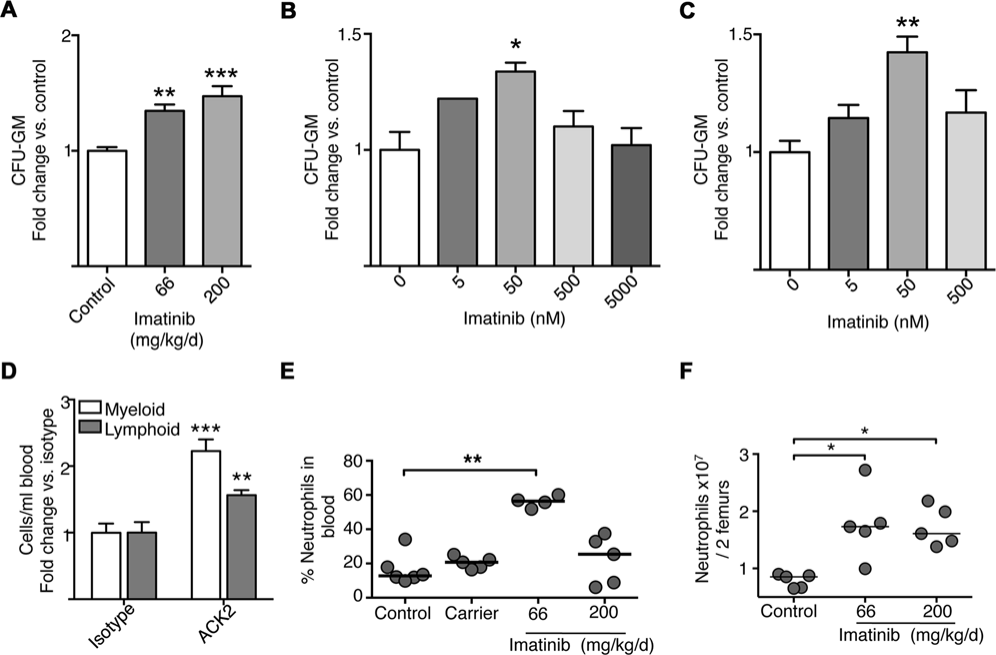
Effects of imatinib on progenitor differentiation in culture, and effects of anti-c-Kit neutralizing antibody. **(A)** Colony forming cell (CFC) assays with bone marrow derived from mice treated with imatinib at 66 or 200mg/kg/d for seven days or left untreated (Control). Bone marrow was cultured in the absence of imatinib, CFU-GM colonies were counted after 10 days. **(B)** CFC assays with bone marrow derived from naïve mice and cultured in the presence of increasing concentrations of imatinib. CFU-GM colonies were counted after 10 days. **(C)** CFC assays with human donor bone marrow cultured in the absence or presence of increasing concentrations of imatinib. CFU-GM colonies were counted after 14 days. All CFU results are presented as a percentage of untreated controls. Results represent combined data from three independent experiments with three replicates per experiment. A Kruskal-Wallis nonparametric test was used to determine significance of each concentration compared to control. **(D)** Effect of anti-c-Kit neutralizing antibody. 3μg of either anti-c-Kit neutralizing antibody (ACK2) or the IgG2b isotype control was administered for five to seven days, and the number of myeloid cells, including neutrophils, monocytes, and eosinophils, and lymphoid cells, including B cells and T cells and NK cells, in the blood determined by flow cytometry. Average fold changes compared to the isotype control on combined myeloid or lymphoid cell populations are presented. **(E**) Mice were treated with imatinib at 66 or 200mg/kg/day or with pumps filled with water for 28 days, the vehicle for imatinib. On day 7, mice were bled from the tail vein and numbers of neutrophils determined by flow cytometry with 6 mice per condition. For all data sets, the line in each group of data points represents the median. **(F)** Effects of dosage on neutrophil and monocyte numbers in bone marrow. Gleevec was administered at 66 or 200 mg/kg/d for 7 days without infection, and neutrophils were enumerated by flow cytometry. A Mann-Whitney nonparametric test was used to determine significance. Data shown are representative of three independent experiments.

To further characterize the effects of imatinib on the expansion and differentiation of myeloid progenitors, bone marrow from imatinib-treated mice was plated in semi-solid media and analyzed by Colony Forming Cell (CFC) assay to detect and quantify colonies of granulocyte-macrophage hematopoietic progenitors (CFU-GM). As shown in Fig. 4A, bone marrow derived from mice treated with imatinib at 66mg/kg/d or 200 mg/kg/d for seven days yielded ~34% more CFU-GM colonies compared to marrow from naïve mice. These data suggest that treatment with imatinib induces an irreversible commitment of HSCs into progenitors that can differentiate *ex vivo* into myeloid cells.

To determine whether cells from naïve animals could likewise be induced to differentiate into myeloid-type colonies when treated with imatinib in culture, CFC assays were performed on naïve bone marrow cultured with various concentrations of drug. As shown in Fig. 4B, addition of imatinib at 50 nM caused a 33% increase in CFU-GM, whereas concentrations exceeding 500 nM, were without effect. We also assessed the effects of PTK inhibitors in CFC assays using marrow derived from human donors. Addition of low concentrations of imatinib (50 nM) to the media maximally increased the number of CFU-GM by 42% compared to untreated marrow (Fig. 4C). By contrast, and in accordance with previous reports [46] concentrations at or exceeding 500nM were without effect. Together, these data suggest that imatinib induced an irreversible differentiation of HSCs or progenitors into myeloid cells in a dose-dependent fashion, and that imatinib effects on myelopoiesis *in vivo* are recapitulated in cultures of murine and human cells *in vitro*.

Partial inhibition of c-Kit affects myeloid and lymphoid cells in the blood. Previous studies suggested that administration of high concentrations (1mg) of the c-Kit neutralizing antibody ACK2 to C57Bl/6 mice ablated hematopoietic progenitors [47]. However, because the effects of imatinib appeared strongly dose-dependent, we reasoned that partial inhibition of c-Kit might recapitulate some or all effects of the drug. To test this hypothesis, we assessed the effects of ACK2 at lower concentrations (0.3ng, 3ng, 3μg and 30μg; Fig. 4D). Increased overall numbers of myeloid cells, including neutrophils, eosinophils and monocytes, were evident in the blood of mice treated with 3 μg of ACK2 (~2 fold on average), relative to an isotype control (Fig. 4D), though the effect was not to the same extent as that seen with imatinib. However, unlike imatinib, ACK2 also increased the numbers of lymphoid lineage cells by ~1.5 fold (Fig. 4D). We were unable to assess effects of ACK2 on numbers of MPPs directly because the antibody interfered with bone marrow staining panels. Nevertheless, low doses of ACK2, which may partially inhibit c-Kit kinase activity or reduce levels of c-Kit protein, appeared sufficient to induce expansion of leukocytes in the blood. Such an effect is consistent with an increase in myeloid and lymphoid progenitors. The observation that ACK2 affects both myeloid and lymphoid cells whereas imatinib predominantly affects myeloid cells suggests that inhibition of other kinases governs myeloid lineage commitment.

We also tested the effect of the ACK2 antibody in the context of infection. Although treatment with the antibody resulted in a ~2-fold decrease in CFU on average (S4C Fig). Although these data did not reach the 0.05 level of statistical significance, they “trended” in the right direction. We surmise that the limited efficacy is due to the fact that the antibody is not as efficient as the drug in inducing myelopoiesis.

### Imatinib-induced expansion of myeloid cells depends on dose

We next assessed numbers of myeloid cells in the blood and bone marrow of uninfected mice treated with imatinib at 200mg/kg/d, a dose previously shown to have no anti-mycobacterial effects. As shown in Fig. 4E, whereas imatinib at 66mg/kg/d increased the percentage of neutrophils in the blood compared to untreated controls from 18% to 60% (see also Fig. 1), with 200mg/kg/d no such increase was evident; similar dosage effects were apparent with monocytes in the blood. Interestingly, in the bone marrow imatinib at 66 or 200mg/kg/d induced comparable increases in numbers of mature neutrophils and monocytes (Fig. 4F). Accordingly, bone marrow derived from mice treated with imatinib 200 mg/kg/d for seven days yielded ~40% more CFU-GM colonies compared to marrow from naïve mice, an increase comparable to that observed with marrow from animals treated with 66mg/kg/d (Fig. 4A). Likewise, comparable increases in numbers of LSKs, HSCs, and MPP2, MPP3 and MPP4 cells were evident with imatinib at doses of 66 and 200mg/kg/d (Fig. 3A-C). Thus, accumulation of mature myeloid cells in the marrow was positively correlated with increases in mature cells in the blood and spleen at 66mg/kg/d, but no such correlation was evident at doses of 200mg/kg/d or higher. Notably, infection plus 200mg/kg/d imatinib did not induce substantial increases in the numbers of myeloid cells in the blood and spleen beyond that seen with infection alone. Together, these data suggest that imatinib facilitates exodus of myeloid cells from the bone marrow only at lower doses, but inhibits this process at higher doses. Because doses of 66mg/kg/d have proven most effective in bacterial infection studies [30], our subsequent analysis focused on effects of the drug at this dose and not at higher doses.

### Imatinib treatment alters CXCR2 expression on neutrophils in the bone marrow

**Fig 5:**
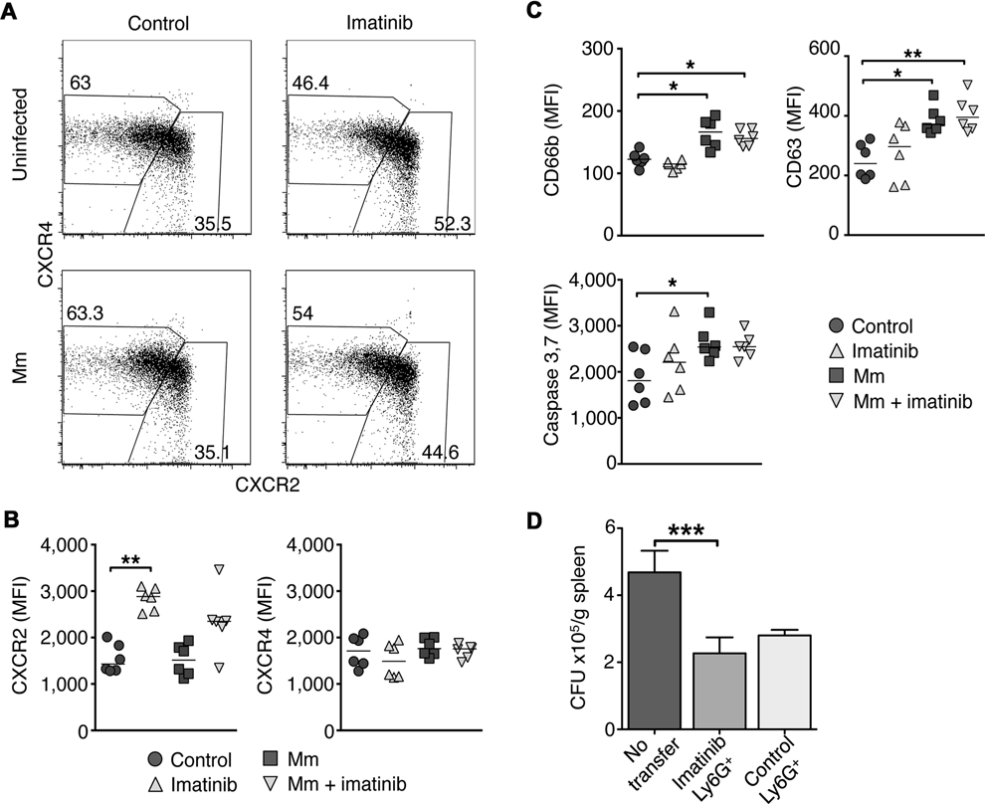
Effects of Imatinib on CXCR2 expression, activation, and apoptosis in neutrophils. C57Bl/6 mice were administered imatinib at 66 mg/kg/d or left untreated. Beginning 24h post-treatment mice were either injected in the tail vein with 10^5^ CFU Mm 1218R or left uninfected. At 7d post-treatment, bone marrow was collected from both femurs. **(A**) Representative flow cytometry plots of CXCR4 and CXCR2 expression on neutrophils from bone marrow using flow cytometry. CXCR2^hi^CXCR4^lo^ and CXCR4^hi^CXCR2^lo^ subsets were identified, and frequencies of total neutrophils are displayed in boxes. **(B)** Median fluorescence intensity (MFI) of CXCR2 and CXCR4 on neutrophils from bone marrow. Cumulative data from two independent experiments are presented with six mice per condition. The line in each data set represents the median. **(C)** C57Bl/6 mice were treated as in **A**. Activation status was assessed by surface expression of CD66b (secondary granules), CD63 (primary granules) and apoptosis by intracellular staining for caspase 3 and 7 activity of the total neutrophils (Ly6G^+^Ly6C^+^) from spleen. Cumulative data from two independent experiments are presented with 6 mice per condition. **(D**) C57Bl/6 mice were administered imatinib at 66mg/kg/d or left untreated. On d7, splenic neutrophils were isolated using Ly6G^+^ microbeads and 4 x10^6^ cells were injected via the left tail vein into each naïve recipient mice, and infected via the right tail vein with 10^5^ CFU Mm. Forty eight hours later, spleens were harvested and CFU/gram were determined. CFU/g spleen from mice injected with carrier, or with neutrophils from imatinib-treated mice or mice treated with carrier (water). Cumulative data from three independent experiments are presented with 15–25 animals per condition. The line represents the median. A Mann-Whitney test was used for pairwise comparisons, and a Kruskal Wallis test for multiple comparisons.

Retention of neutrophils within the bone marrow is correlated with expression of CXCR4 on the cell surface [48], whereas migration of neutrophils into the blood and to peripheral sites is associated with surface expression of CXCR2 [48–50]. Treatment with imatinib at 66mg/kg/d increased the percentage of CXCR2^hi^CXCR4^low^ neutrophils by 17% in uninfected mice and by 9% in infected mice, compared to control animals (Fig. 5A). Accordingly, imatinib increased the median fluorescence intensity (MFI) of CXCR2 on neutrophils by 1.8-fold and 1.5-fold in uninfected and infected mice, respectively (Fig. 5B). Thus, imatinib at 66mg/kg/d increased the surface expression of CXCR2 and the proportion of CXCR2^hi^CXCR4^low^ neutrophils in the bone marrow. These data are consistent with an increased capacity of neutrophils to migrate from the bone marrow to the blood at 66mg/kg/d, and with the observed increase of neutrophils in the blood at this dose.

### Neutrophils from imatinib-treated mice are not intrinsically activated, but become activated and undergo apoptosis in response to Mm infection

Proinflammatory stimuli cause neutrophils to become activated and mobilize secondary and primary granules, which fuse with the plasma membrane and release antimicrobial compounds [51]. Neutrophil degranulation can be quantified by the surface expression of CD66b, a marker for secondary granules, or CD63, a marker for primary granules (Fig. 5C). In naïve mice, imatinib at 66 mg/kg/d did not alter the surface expression of CD66b or CD63 on neutrophils in the bone marrow, blood or spleen, indicating that, although the neutrophil numbers increased, the neutrophils were not activated (Figs. 5C and S5A). However, in the context of a Mm infection, neutrophils from control animals or animals treated with imatinib displayed increased surface expression of CD66b and CD63 relative to uninfected controls (Fig. 5C). This effect was most pronounced in the spleen, the site of the greatest concentration of bacteria [30]. Together, these data suggest that neutrophils are not activated by imatinib at low doses, but retain the capacity to become so upon infection.

Following activation or phagocytosis, neutrophils undergo apoptosis [52], which is characterized by cleavage of pro-caspase-3 or -7 (caspase-3/7) into enzymatically active forms [53]. Neutrophils from mice treated with imatinib at 66 mg/kg/d did not display significant differences in levels of active caspase-3/7 compared to untreated mice (Figs. 5C; S5A). However, neutrophils in the spleens of Mm infected mice showed significantly elevated levels of caspase-3 and 7 whether treated with imatinib or not (Figs. 5C; S5A), in accordance with reports that neutrophils become activated in response to mycobacterial infection [52]. These data indicate that imatinib does not alter apoptosis in activated neutrophils.

### Increased numbers of neutrophils are sufficient to reduce Mm bacterial load

To determine whether an increase in neutrophil numbers alone was sufficient to reduce bacterial load following infection with Mm, adoptive transfer experiments were performed. Neutrophils were purified from either control or imatinib-treated animals. Recipient mice were then injected with 4x10^6^ neutrophils derived from either control or imatinb-treated animals, and then infected with Mm. This number of transferred neutrophils represented less than half that found in the spleens of animals treated with imatinib; however, levels equivalent to that with drug could not be achieved because purifying larger numbers proved unfeasible. Bacterial load in infected organs was assessed 48h after adoptive transfer and infection. As shown in Fig. 5D, increasing the overall number of neutrophils, whether derived from PBS- or imatinib-treated mice, reduced CFUs in the spleen by ~2-fold, and no significant difference was evident between neutrophils derived from control or imatinib-treated animals. Thus, increasing neutrophil numbers alone is sufficient to reduce mycobacterial load, and imatinib does not augment the specific killing capacity of neutrophils. Notably, other myeloid cell types that increase in number with imatinib may also contribute to the observed reduction in CFU. Moreover, despite administration of sufficient anti-Ly6G antibody (1A8; [54]) to saturate binding sites on neutrophils in imatinib-treated mice, only 30–40% of the neutrophils could be depleted (S5B and S5C Figs). These data suggest that administration of high concentrations of 1A8 under neutrophilic conditions saturates cellular removal mechanisms. Because such mechanisms may also contribute to removal of infected cells, and because only a fraction of the cells can be depleted, using such methods to evaluate the role of neutrophils in imatinib-mediated reduction of CFUs has proven untenable.

### Imatinib reduces bacterial load in mice infected with pathogenic *Francisella* species

**Fig 6:**
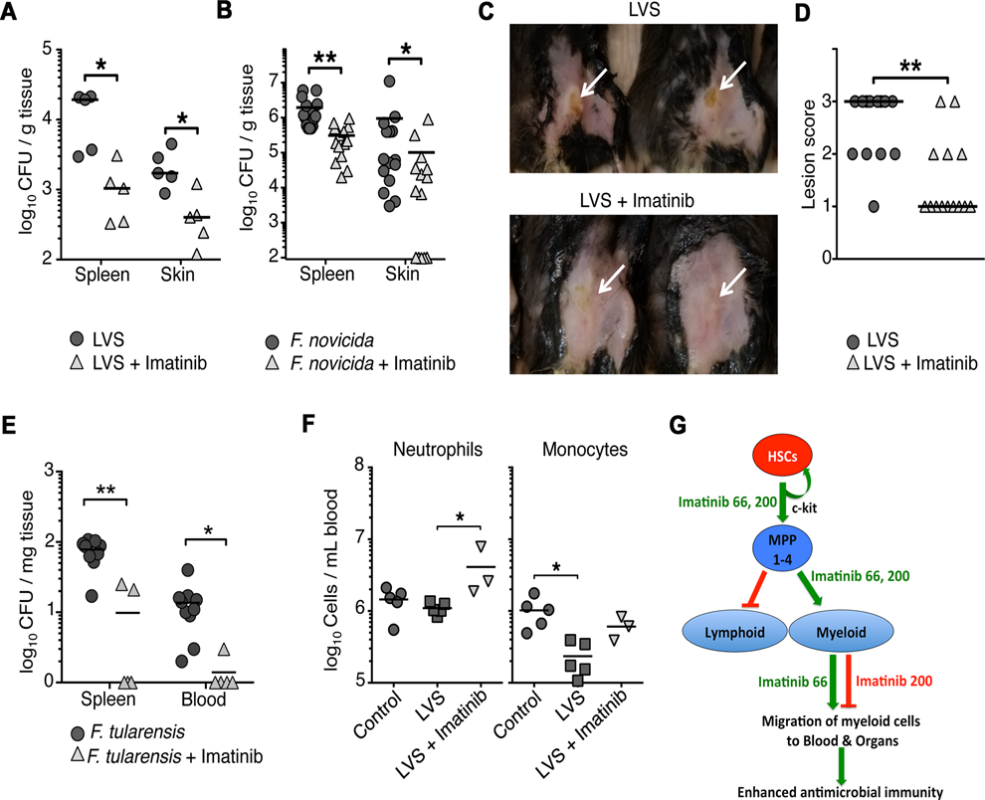
Imatinib decreases bacterial load of pathogenic *Francisella spp*. *in vivo*. **(A-B)** C57Bl/6 mice were treated with imatinib at 66 mg/kg/d or water for 7d prior to infection and for the duration of the experiment, and then injected subcutaneously with either ~6x10^6^ Fn **(A)** or ~2x10^5^ LVS **(B)**. After 48h for Fn or 5d for LVS, skin and spleens were collected and CFU/gram of tissue was determined. The limit of detection of the assay was 100 CFUs. **(C)** Images of lesions on control or imatinib-treated mice infected with LVS. Hair was removed from injection site. The lesions are indicated by white arrows. **(D)** Quantitation of lesions from control or imatinib-treated mice. Lesions were scored on a scale of one to three, with a score of one denoting no visible lesion, two a visible lesion but without a break in the skin, and three an open wound. 15 animals were scored per condition. **(E)** Four days following infection with strain Ft Schu S4, CFU/gram of tissue was determined. The limit of detection of the assay was 100 CFU/gm for Ft. The line in each data set represents the median. A Mann-Whitney nonparametric test was used to determine significance. Combined data from three independent experiments are shown. **(F)** Effects of imatinib at 66mg/kg/d on neutrophils and monocytes upon infection with LVS. Data from a representative experiment are shown. **(G)** Summary of effects of Imatinib on myelopoiesis and migration of mature myeloid cells to the blood and organs. Imatinib at doses of 66 or 200 mg/kg/day induces activation, expansion, and maturation of hematopoietic stem cells (HSCs) and multipotent hematopoietic progenitors 1–4 (MPP1-4) in the bone marrow. Imatinib at these doses also stimulates a lineage determination step that induces MPPs to differentiatiate into myeloid precursors and then mature cells (green arrow), but not into cells of the lymphoid lineage (red arrow). Data with low doses of neutralizing antibodies against the c-Kit tyrosine kinase support the hypothesis that effects on HSCs and MPPs are mediated by partial inhibition of c-Kit, whereas effects of the drug on lineage determination appears to be independent of c-Kit. Ultimately, imatinib increases the numbers of mature myeloid cells in the bone marrow, an effect seen with drug alone; the accumulation of mature cells is not evident with the drug in context of infection, which itself appears to mobilize migration of mature cells from the bone marrow to the periphery. Notably, increased numbers of mature cells are only evident in the blood and tissues at lower doses (66mg/kg/d), suggesting that at low doses, Imatinib induces migration of mature cells out of the bone marrow; alternatively, the drug may inhibit migration at higher doses (200mg/kg/d). Importantly, Imatinib mimics the “emergency response” to infection, which likewise induces HSC activation, MPP expansion, myelopoiesis, and migration of mature myeloid cells from the bone marrow to the periphery (green arrows).

Myeloid cells, and particularly neutrophils, are required to contain infections caused by a variety of pathogenic bacteria [55–57]. The observation that imatinib dramatically increased myeloid cell numbers led us to ask whether the drug might be effective against other bacterial infections, which, unlike mycobacteria [30,33], do not utilize Abl or other imatinib-sensitive kinases for pathogenesis. Growth and intracellular survival of the *Francisella* species *F*. *novicida* (Fn) and *F*. *holarctica* (LVS, the live vaccine strain), in either broth or in macrophages remained insensitive to imatinib (S6A-S6D Fig). Because these bacterial strains are lethal in mice within a few days of infection, imatinib was provided at 66 mg/kg/d for one week prior to infection with Fn or LVS, and throughout the course of infection (48hrs for Fn and 5 days for LVS). Imatinib reduced Fn and LVS CFU in the spleen and skin of infected animals by up to 10-fold compared to untreated animals (Fig. 6A,B). In addition, pathology at the site of infection with LVS was assessed. Lesions in mice treated with imatinib were either reduced in size or absent compared to controls (Fig. 6C,D). Imatinib was likewise effective against *F*. *tularensis* (Ft), reducing CFUs in blood and spleen by on average 8-fold and 15-fold, respectively (Fig. 6E). By contrast, imatinib at a dose of 200mg/kg/d was without effect on CFU (S6E Fig). Unlike Mm, *Franciscella* infection did not activate a strong emergency response, and appeared to suppress immune cell numbers. Thus, with LVS infection, numbers of neutrophils remained constant, but numbers of monocytes, B, T, and NK cells decreased (Figs. 6F and S6F), perhaps reflecting a partial suppression of immune function or killing of infected cells by the bacteria. With infection plus imatinib (66mg/kg/d), numbers of neutrophils and monocytes increased, although only the neutrophil increase reached statistical significance (p<0.05); imatinib was without effect on T, B, or NK cells (S6F Fig). Thus, imatinib may counter myelosuppressive effects of *Franciscella* infection by increasing myelopoiesis, or decreasing bacterial CFU, or both. Moreover, these data suggest that imatinib may provide a protective effect against a broad range of pathogens, including those whose intracellular survival does not depend on the activity of Abl1 and other imatinib-sensitive kinases.

## Discussion

Several lines of evidence suggest that imatinib, at the low doses used in this study, activates dormant HSCs, which rapidly differentiate into MPPs and mature myeloid cells. Low levels of imatinib did not cause accumulation of HSCs nor augment their transplantability. Nevertheless, the drug reduced accumulation of HSCs and MPP1 and MPP2 in infected mice (Fig. 3B,C), suggesting that the drug facilitates flux of early stem cells and progenitors into more differentiated cell types.

Data presented here suggest that partial inhibition of c-Kit may result in expansion of HSCs and/or MPPs but not their differentiation. Thus, low doses of anti-c-Kit mAb ACK2 increased numbers of cells of both myeloid and lymphoid origin (Fig. 4D), whereas imatinib appeared to both expand HSCs and MPPs, and direct MPPs predominantly towards myeloid lineages (e.g. Figs. 1–3). HSCs express c-Kit, and c-Kit ligand both promotes self-renewal of HSCs and maintains quiescence [58–61]. In accordance with our observations, G-CSF, which mobilizes HSCs and augments granulopoiesis [41], also induces the production of proteases that cleave c-Kit and its ligand, thereby reducing c-Kit activity [62]. Finally, mice with mutations in *kit* regulators (C57BL/6J-Kit^W-sh^) display both increased numbers of myeloid cells in the bone marrow and peripheral neutrophilia [63]. However, because these mice contain more than thirty other mutations, it has not been possible to ascribe these effects to c-Kit directly.

Recent transplantation experiments have highlighted the importance of c-Kit surface expression and signaling levels in regulating self-renewal of HSCs (c-Kit^lo^) versus their differentiation (c-Kit^hi^), with the c-Kit^lo^ cells giving rise to c-Kit^hi^ cells, but not vice versa [61]. c-Kit^hi^ HSCs in turn support long-term lympho-myeloid grafts, although they exhibit a bias towards the megakaryocytic lineage [61]. Our data with the low doses of ACK2 antibody suggest that partial inhibition of c-Kit may govern transition of activated HSCs into MPPs and expansion of MPPs. However, the observation that low doses of the ACK2 antibody induce increases in both myeloid and lymphoid cells suggests that lineage determination by imatinib is regulated by kinases other than c-Kit. In this regard, inhibition of PDGFR, also an imatinib target, has been associated with differentiation of megakaryocytes [64]. Imatinib may cause differentiation of myeloid lineage cells by either inhibiting lymphoid differentiation, or alternatively, augmenting myeloid differentiation, perhaps via effects on lymphoid-myeloid progenitors distal to MPPs [42,43].

Finally, although accumulation of MPPs and mature myeloid cells in the bone marrow was evident at doses of 66 and 200mg/kg/d, accumulation of myeloid cells in the blood occurred only at the lower dose (Figs. 3 and 4). Surface expression of CXCR2 increased on mature neutrophils in the marrow in animals treated with 66mg/kg/d, consistent with an increased capacity to migrate out of the marrow. Notably, even with infection, fewer myeloid cells were evident in the periphery at high doses compared to low doses. Thus, whereas low doses of imatinib facilitate exodus of myeloid cells out of the bone marrow, higher doses appear to inhibit this process.

As summarized in Fig. 6G, imatinib doses of 66mg/kg/d appear to regulate hematopoiesis at three distinct steps: (i) flux of HSCs and MPPs, (ii) maturation of myeloid but not lymphoid progenitors and (iii) migration of mature myeloid cells to the blood and peripheral organs. At higher doses (200mg/kg/d) migration appears inhibited. In all these ways, imatinib at doses of 66mg/kg/d mimics “emergency hematopoiesis” [65], a natural response to infection that results in increased numbers of circulating myeloid cells, particularly monocytes and neutrophils. Notably, at optimal doses, the effect of imatinib on myelopoiesis appears more pronounced than that seen with infection (Figs. 1A, 3, 4).

Differences in half-life of imatinib (~1.5 hrs in mice versus ~15 hours in humans) preclude direct comparisons of applied doses between the two species, However, comparisons can be made by considering steady state levels of the drug. In mice, imatinib at doses of 66mg/kg/d yields steady state levels in the serum of ~57ng/ml (~100nM), which corresponds to less than 5% of that observed in CML patients treated with the minimal clinical dose of imatinib at 400mg QD (~1500ng/ml trough to 3000 peak, or 2.5–5μM)[37,38]. Thus, increased myelopoiesis seen *in vivo* in mice and *in vitro* using cultured murine and human bone marrow cells (Figs. 1,2, and 4), would likely not be evident at doses currently prescribed for CML patients. Indeed, myelo-suppression has been most commonly associated with doses higher than 400mg QD or with long-term administration of the drug in people [66]. However, it is noteworthy that in rare instances, GIST patients exhibit a dermal rash called Sweet’s syndrome [67], which is characterized by a localized neutrophilia. The rash abates when the drug is discontinued. It remains possible that individuals who contract Sweet’s syndrome are either non-compliant with the treatment regimen, or rapidly metabolize imatinib, either of which could result in lower levels of drug in the blood. In addition, Druker and colleagues noted in their initial clinical study with imatinib that half of those assigned to receive 25, 50, or 85 mg imatinib QD were removed from the study within two months because of elevated white-cell or platelet counts, which required therapy prohibited by the protocol [68]. Although not definitive because of the underlying leukemia, these doses are at the upper range we would expect might cause induction of myelopoiesis, and taken together with our data showing increases in CFC numbers with human marrow at concentrations of 5–50 nM (Fig. 4), suggest that this effect will be evident in humans, a prospect we are currently testing.

Our results raise the possibility that imatinib may be useful in treating several conditions associated with dysregulation of neutrophil homeostasis, which result in increased risk of severe infection. These include hereditary disorders such as cyclical neutropenia, cancer, autoimmune diseases, microbial infections and myelosuppressive chemotherapeutics. Administration of G-CSF mitigates neutropenia in a variety of conditions [69–71] by stimulating the production of neutrophils in the marrow and their migration into the blood [72]. However, G-CSF has been associated with deleterious outcomes against infections. For example, G-CSF blunts helper T cell responses and increases regulatory T cell responses and recapitulates a “super-shedder” phenotype characterized by excretion of high levels of *Salmonella* and hyperinflammation [73]. Imatinib, by contrast, has the opposite effect, causing reductions in bacterial load [30], decreased regulatory T cell responses, and augmented antigen presentation [36,74,75]. The difference may be that imatinib induces myelopoiesis and an overall increase in all myeloid cells, whereas G-CSF only induces granulopoiesis and neutrophilia, or, alternatively, that G-CSF has additional effects on other cell types [71]. Thus, imatinib may be useful for patients suffering neutropenia and have fewer deleterious side effects than G-CSF, a prospect we are currently testing.

We have proposed that imatinib may be useful in treating a broad range of infections caused by bacterial and viral pathogens that use Abl1, Abl2 or other imatinib-sensitive PTKs for pathogenesis [76]. These include, for example, poxviruses, filoviruses (Ebola), and Mtb [23,28,30–32,76,77]. Different dosing strategies may apply to different pathogens depending on the mechanism of action. Thus, for poxviruses and filoviruses, imatinib effects likely depend on inhibition of viral dissemination, which requires Abl-family kinases [23,31,32]. Notably, the optimal dose for inhibiting Abl-family kinases and treating poxvirus infections with imatinib is 200mg/kg/d, a dose that stimulates myelopoiesis but without attendant increases in myeloid cell numbers in blood and spleen.

By contrast, other pathogens, particularly those that trigger antimicrobial responses mediated by neutrophils and macrophages, may be more susceptible at lower doses of imatinib. Mycobacteria infections are optimally responsive to imatinib at 66mg/kg/d [30], a dose that triggers both myelopoiesis in the marrow, and increases in myeloid cells in blood and spleen. In acute Mtb infections, macrophages, followed by neutrophils, transiently increase in numbers in the lungs, reaching the highest levels just prior to arrival of DCs [54]. Both neutrophils and macrophages become infected, and depleting neutrophils increases the frequency of Mtb-infected DCs in the lungs, but decreases trafficking of DCs to the mesenteric lymph nodes, which precludes DC-initiated adaptive responses [54]. Thus, neutrophils may promote adaptive responses to Mtb by delivering bacterial antigens to DCs in a way that enables DC migration, and allows more effective antigen presentation and activation of naive CD4 T cells. Other evidence suggests that the anti-infective state induced by imatinib in the host and mediated by myeloid cells, resembles that seen in human patients who are protected from TB. First, studies of initially IGRA-negative TB household contacts indicate that low baseline neutrophil count is a predictor of subsequent IGRA conversion [78]. Thus, protected individuals may have, by virtue of continuous or repeated exposure, a heightened basal myeloid response that provides protection, and which resembles that induced by imatinib. Moreover, Kaushal and colleagues have shown in primates that mutants of Mtb, which are cleared by the immune response, induce a strong hematopoietic response, whereas Mtb does not [79]. Thus, Mtb may suppress the emergency response, which may be overcome by imatinib. Finally, Fletcher and colleagues have shown that BCG vaccinees who remain unprotected from TB have transcriptional signatures that may be indicative of either low myeloid responses or hyperactive ones, whereas protected individuals have an intermediate response (H. Fletcher, personal communication). Current efforts are aimed at determining whether these protective responses resemble those seen with imatinib.

Like Mtb, resolution of *Franciscella* infections depends on neutrophils [57], and Franciscella appears to suppress both the emergency response (Fig. 6F), and adaptive responses (S6F Fig). Our observations suggest that *Francisella spp*. do not require imatinib-sensitive kinases for pathogenesis *in vitro*, yet are still susceptible to imatinib *in vivo* (Figs. 6 and S6). Together, our data raise the possibility that imatinib may have utility against a wide range of pathogens that do not necessarily utilize Abl-family kinases for pathogenesis, by overcoming pathogen strategies to limit or subvert the emergency response. Moreover, agents such as imatinib may even be efficacious against strains resistant to conventional antibiotics, and may even act synergistically with co-administered antibiotics, a result suggested by our previous studies [30].

To realize the potential of imatinib as an immunomodulatory therapeutic for Mtb infections will require a balanced inflammatory response, without favoring hyper- or hypo-inflammation, which have been shown to be deleterious (e.g. [80–83]). Notably, the hematopoietic response generated by imatinib is titratable with dose. This response comprises an increase in numbers of all myeloid cells, thereby providing a limit on inflammation [84], rather than an increase in a single cell type, such as neutrophils, which can by themselves induce significant damage [85]. Nevertheless, heterogeneity in immune response (e.g. [82]) or disease stage could affect how an individual responds to up-regulation of the emergency response. Thus, careful dosing regimens and treatment at appropriate disease stages, in conjunction with assessments of diagnostic biomarkers and clinical signs will be required to ensure optimal activity of the drugs with minimal toxicity. A more complete discussion of the promise and caveats associated with host directed therapeutics for TB, including imatinib, as well as trial design and measures or efficacy, is reviewed elsewhere by one of us (D.K.;[77]).

In summary, we demonstrate a surprising immune-stimulatory effect of imatinib on myelopoiesis, which depends in part on c-Kit and occurs at subclinical doses. These observations have important implications for the use of imatinib as an immunostimulatory therapeutic against neutropenia and against infectious pathogens, including those that do not utilize host imatinib-sensitive kinases for pathogenesis.

## Materials and Methods

### Flow cytometry

For analysis of immune cells in whole blood, bone marrow and collagenase-digested splenocytes [86] were incubated with blocking mAb 2.4G2 anti-FcγRIII/I and live/dead probe (Alexa Fluor 430; Invitrogen, Grand Island, NY). Cells were labeled with CD11b (M1/70), B220 (RA3-6B2), and Ly6C (AL-21) antibodies from BD Biosciences (San Jose CA), CD19 (MB19-1), Thy1.2 (53–2.1), F4/80 (BM8), CD11c (N418), CD8α (53–6.7), Gr-1 (RB6-8C5) from eBioscience (San Diego, CA) and NK1.1 (PK136) from BioLegend. Cells were then stained with Streptavidin (QDot655; Invitrogen) before fixation.

To isolate neutrophils for activation and degranulation assays, blood, bone marrow and spleen cell samples were processed on ice and in PBS-EDTA buffer to prevent activation of the cells. Bone marrow and spleen cells were homogenized and then filtered. Then, cells were incubated with blocking mAb 2.4G2 anti-FcγRIII/I (BD Biosciences) and live/dead probe (Yellow; Invitrogen) along with labeled CD11b (M1/70), B220 (RA3-6B2), Ly6C (AL-21), and CD66b (G10F5) antibodies from BD Biosciences, Ly6G (1A8), CXCR2 (TG11), CD63 (MEM-259) from BioLegend (San Diego, CA), CXCR4 (TG12) from eBiosciences and FLICA probe for caspases 3/7 from Novus Biologicals (Littleton, CO). Notably, there is good cross-species reactivity with mouse neutrophils with the anti-human CD66b antibody (clone G10F5; [87]). All samples were acquired on a BD Biosciences LSR II (BD Biosciences and analyzed using FlowJo (TreeStar, Inc; Ashland, OR).

To deplete neutrophils in naïve or imatinib treated mice, 300ug of anti-Ly6G antibody clone 1A8 or 2A3 isotype control were administered one day prior to imatinib treatment and 2 days post treatment as described previously [54]. Blood was collected on the third day after imatinib treatment and neutrophil numbers were determined by flow cytometry. Both fluorescently labeled anti-Ly6G (clone 1A8) and anti-Gr-1 (specific for Ly6G and Ly6C) antibodies were unable to bind some neutrophils from 1A8-treated naïve or imatinib-treated mice owing to continued presence of 1A8 depleting antibody occluding the epitope shared by both antibodies. Thus, full enumeration of blood neutrophils was achieved with an anti-DEC-205 fluorescently labeled antibody in conjunction with an anti-Ly6C antibody. Neutrophil depletion of mice with 1A8 reduced median numbers of blood neutrophils to ~6% of the number seen in naïve animals, in line with previous reports [54]. However, depletion of imatinib-treated mice reduced numbers to only 62% of that seen in naïve animals or to 20% of that seen in imatinib-treated animals. Thus, our data suggest that in the presence of imatinib, the maximum number of neutrophils possible were depleted with 1A8, and increasing the concentration of 1A8 would not deplete more, and therefore that the mechanism by which neutrophils were removed from circulation appeared to be saturated in the presence of imatinib plus 1A8. Together these data suggest that we could not efficiently deplete neutrophils under these conditions. Moreover, because of this inefficiency and because cellular depletion mechanisms likewise are required to remove infected cells, we could not evaluate whether neutrophils were necessary for imatinib-mediated reduction of CFU.

To identify LSK, HSC, and multipotent progenitor populations (MPP1-4), bone marrow was flushed from femurs with DMEM plus 10% fetal bovine serum and labeled with the following mAbs: CD34 (HM34) and erythroid cells (TER-119) from Biolegend, CD135 (A2F10) from eBiosciences, sca-1 (D7), c-Kit/CD117 (2B8) and the following biotinylated mAbs (CD19 lineage (ID3), NK1.1 (PK136), B220 (RA3-6B2), GR1 (RB6-8C5), CD11b (M1/70), CD19 (1D3), CD4 (GK1.5), CD8 (53–6.7) CD150 (Q38-480) CD48 (HM48-1), followed by a secondary stain with streptavidin, from BD Biosciences. Cell numbers are expressed as per two femurs based on cell counts or, alternatively fluorescent beads to measure the concentration of cells during flow cytometry. Both methodologies yielded similar numbers of cell subsets.

### FACS sorting of LSK cells

Bone marrow was flushed from donor femurs with sterile PBS containing 1% heat-inactivated fetal calf serum (PBS/FCS). Bone marrow cells were stained with a cocktail of antibodies from BD Biosciences: biotinylated lineage 1 antibodies (CD3, CD11b, CD19, CD49b, IgM, and Ter119), PE-conjugated lineage 2 antibodies (CD4, CD8, GR-1, and I-A^b^), as well as, B220 PE-Cy5, c-kit APC, and Sca-1 PE-Cy7, followed by streptavidin APC-Cy7. For some experiments, cells were sorted using a FACS-Aria cell sorter and data analyzed using Diva Version 5.1 software (both from BD Biosciences). After initial scatter-based gating to exclude doublets, the B220^−^ population was further gated to identify and sort the lineage^-^ Sca-1^+^ c-Kit^+^ (LSK) cell population that contains hematopoietic stem cells [44].

### Bone marrow colony-forming cell assays

Murine bone marrow (BM) cells were flushed from femurs and tibias using Iscove’s MDM (Stem Cell Technologies; Vancouver, BC, Canada) containing 2%FBS. Cells were triturated and filtered through a nylon screen to obtain a single-cell suspension. Human BM was obtained by aspiration from the posterior iliac crest in an IRB-approved protocol that enrolled normal volunteers (see below) and was depleted of RBCs by treatment with 0.8% Ammonium Chloride Solution (Stem Cell Technologies) according to manufacturer’s instructions. BM was plated in duplicate (2 × 10^4^ nucleated cells/35 mm dish for murine BM, 5 x 10^4^ nucleated cells/35 mm dish for human BM) in semisolid methylcellulose medium containing stem cell factor (SCF), interleukin-3 (IL-3), erythropoietin (Epo), and either interleukin-6 (IL-6) for murine BM (MethoCult M3434, Stem Cell Technologies), or granulocyte/macrophage colony stimulating factor (GM-CSF) for human BM (MethoCult H4434, Stem Cell Technologies), plus or minus the indicated concentrations of imatinib. Plates were incubated at 37°C, 5% CO_2_ and >95% humidity and Granulocyte-Macrophage colonies (CFU-GM) were identified by morphology and counted after 10–12 (murine) or 14–16 (human) days of incubation.

### Competitive BM transplant experiments

In the first experiment, 2x10^6^ bone marrow cells from naïve CD45.2 C57Bl/6 mice or CD45.2 C57Bl/6 mice treated with imatinib for 7 days were mixed with 2x10^6^ bone marrow cells from untreated CD45.2 GFP-C57Bl/6 mice, and either the naïve-GFP BM mixture or the imatinib-GFP BM mixture injected I.V. into the tail vein of a congenic CD45.1^+^ C57Bl/6 recipient mice. At four and twelve months post-transplantation, mice were bled and the relative numbers of WBC derived from GFP animals, or from either naïve- or imatinib-treated donors, or recipient mice were analyzed by flow cytometry, taking advantage of the GFP label and CD45.1 congenic marker. In the second experiment, 5000 FACS-sorted LSK cells from naïve- or imatinib-treated mice were co-transplanted with 300,000 untreated BM cells from GFP^+^ mice. The relative numbers of WBC derived from GFP, or either naïve- or imatinib-treated donors, or recipient mice were determined 4 to 12 months post-transplantation.

### Delivery of drugs *in vivo*

For experiments with imatinib, the mesylate salt was dissolved in water and loaded into Alzet pumps (Braintree Scientific, 1007D or 2002; Cupertino, CA) capable of dispensing a continuous flow of drug at doses ranging from 1, to 300mg/kg/day. Pumps were inserted subcutaneously into anesthetized 6-week old male C57Bl/6 mice (Jackson Laboratories; Bar Harbor, ME). At all doses tested, no weight loss or other adverse events were evident in uninfected animals. Alzet pumps were inserted 24 h to 7 days prior to manipulation or infection, and drug delivery was maintained for the duration of the experiment (7 to 28 days).

Some variation in CFU in Mm infection, or with plaque forming units (PFU) with vaccinia virus was evident when using different sources or lots of drug. The drug lot used in this study achieved a steady state serum concentration in the blood of 57+/-21ng/ml at 66mg/kg/d, and 165ng/ml +/- 66ng/ml at 200mg/kg/d. Discrepancies between lots or sources were accounted for by the fact that different lots of drug applied at the same dose sometimes yielded different steady state concentrations of drug in the blood; however, no phenotypic differences were evident when animals were dosed such that serum levels were equivalent. All the data shown in this paper used a single lot of drug, but the phenotypes have been reproduced with different lots from different manufacturers. Together, these data highlight the utility of normalizing phenotypes to the steady state concentration of drug in the blood rather than to the administered dose.

### c-Kit neutralization *in vivo*

Mice were injected intravenously with 3μg of c-Kit neutralizing antibody ACK2 (eBiosciences), or 3μg of IgG2b isotype control (eBiosciences), every 48h for seven days at the indicated concentrations. Blood was collected from mice at day seven and the number of myeloid cells and lymphocytes was determined by flow cytometry.

### Bacterial strains and *ex vivo* assays

*M*. *marinum (Mm)* strain 1218R (ATCC 927), a fish outbreak isolate, was grown in Middlebrook 7H9 broth (7H9) (BBL Microbiology Systems, Cockeysville, MD) supplemented with ADC (Difco Laboratories, Detroit, MI,) and 0.05% Tween 80 (Mtb) (Sigma-Aldrich, St. Louis, MO) or 0.025% Tween 80 (Mm). For CFU assays 7H10 agar supplemented with 10% oleic acid-albumin-dextrose-catalase (OADC) was used (Difco Laboratories, Sparks, MD). For *in vivo* Mm infections, bacterial stocks were grown at 30°C for 2 days to an OD_600_ of 0.4 (Eppendorf, BioPhotometer; Hamburg, Germany), the cells were diluted with PBS to 10^5^ CFU/100ul. *F*. *novicida* (Fn) strain U112 overnight cultures were grown at 37°C with aeration in tryptic soy broth (TSB; Difco) supplemented with 0.02% L-cysteine (Sigma-Aldrich) while LVS cultures were grown in modified Mueller-Hinton broth (mMHB) supplemented with 1 mM CaCl_2_, 1 mM MgCl_2_, 0.1% glucose (Sigma-Aldrich), 2% Isovitalex (Difco), and 0.025% ferric pyrophosphate. For macrophage CFU assays, Fn was plated for enumeration on tryptic soy agar (TSA; Difco) and supplemented with 0.01% L-cysteine. Mouse macrophage cell line J774A.1 (ATCC TIB-67) was maintained in Dulbecco’s modified Eagle Medium (DMEM). For *in vivo* CFU assays, Fn experiments were plated on modified Mueller Hinton (mMH) (Difco/BD) plates supplemented with 0.025% ferric pyrophosphate (Sigma-Aldrich), 0.1% glucose, and 0.01% L-cysteine. For both macrophage and *in vivo* assays, LVS was plated on mMH agar supplemented with 2% Isovitalex.

### *In vivo* bacteria infection assays

For *M*. *marinum* infections, six-week old male C57Bl/6 mice were injected in the tail vein with active growing cultures at ~10^5^ CFU/mouse. The number of bacteria injected for each experiment was determined by retrospective plating and was ~2.5x10^5^ CFU/mouse. Seven days after infection, blood, spleen and bone marrow were harvested. For CFU, spleens were weighed and homogenized in a Tissuemise (Fisher Scientific, Hampton, NH) in 1 ml PBS. Each homogenate was diluted and spread on 7H10 agar. Colonies were scored after seven days of incubation at 30°C. Total weight of the organ and colonies per ml of the homogenized organ were used to determine CFU/gram. For *Francisella* infections, C57Bl/6 mice were infected with ~6x10^6^ *F*. *novicida*) or ~2 x 10^5^ *F*. *holartica* (LVS strain), or *F*. *tularensis* (Ft; strain Schu S4). After 48 hours with Fn, or 5 days with LVS, of 4 days with Ft mice from both types of infections were sacrificed and the spleen, liver, and skin at the site of infection were harvested, homogenized, plated for CFU on MH plates, and incubated overnight at 37°C.

### Neutrophil purification and adoptive transfers

Neutrophils were purified from the collagenase-digested spleens derived from control or imatinib-treated mice. Splenocytes were first depleted of B cells with anti-CD19 coated microbeads (Miltenyi; San Diego, CA) then neutrophils were positively selected by anti-Ly6G^+^ microbeads. Purity was assessed on the Ly6G^+^ enriched fraction using the parameters listed above. Neutrophils, defined as CD11b^+^Gr-1^hi^Ly6C^low^. Ly6G^+^ fractions were 100% pure from imatinib-treated mice and 80% pure from naïve mice. We routinely purified ~1x10^6^ neutrophils from the spleen of a naïve mouse and ~8x10^6^ from the spleen of an imatinib treated mouse. For adoptive transfers, 4x10^6^ neutrophils were injected into the left lateral tail vein of naïve recipients. Immediately following the adoptive transfer, 10^5^ Mm were injected into the right lateral tail vein of the mouse. Spleens were harvested 48 hours after the neutrophil transfer and infection, and bacterial CFU determined as described above.

### Histology and immunofluorescence microscopy

Imatinib-treated (66.7mg/kg/day) or water control pumps were implanted one day prior to infection. Day 7 post-treatment, spleens were harvested, frozen in optimum cutting temperature (OCT) compound, cut into 6μm sections, mounted on slides, and then fixed with %100 acetone for 10 min at −20°C. Following rehydration in PBS, slides were permeabilized with PBS +0.5% Triton X-100 +1% BSA, blocked with normal rat serum and anti-FcRIII/I antibodies and then incubated with anti-CD169-FITC (Serotec; Oxford, UK), CD11b-PE (eBioscience), and F4/80-biotin (eBioscience). Tissues were incubated with Streptavidin-APC and mounted with Prolong-GOLD (with DAPI) (Invitrogen). Images were captured using the x10 objective on a Zeiss Axioscope (Carl Zeiss, Germany) and analyzed using ImageJ (National Institute of Mental Health) and DoubleTake (Echo One, Denmark) software. Bone slices from naïve mice or mice treated with imatinib at 66 mg/kg/d and stained with Giemsa and imaged on a Zeiss 200M microscope at x100 or x400 magnification.

### Statistical analysis

Statistical analysis was done using either of two non-parametric tests including the Mann-Whitney test to compare two samples, or the Kruskal Wallis test to compare multiple subsets within a group (e.g. with or without infection and with or without drug). In both tests, the data were pooled and the values ranked. The statistic calculates the probability that the observed ranks of a subset of observations represent a random sampling from the population as a whole, or a significantly different population compared to the group as a whole. Values less than or equal to 0.05 were considered statistically significant. For comparing amongst averaged data from several experiments, a t-test was used to determine significance.

### Ethics statement

For some experiments, bone marrow was obtained from normal human volunteers enrolled in an institutional observational study conducted at the Emoryt transplant center to evaluate the central immune response in healthy volunteers. Samples used in this paper were from normal bone marrow donors who did not have signs of disease including malignancy nor gastrointestinal infection (viral, bacterial, fungal, protozoal) within two weeks of the day of collection. Samples used in this paper were obtained from bone marrow unused for other aspects of the study. The protocol was reviewed by the Emory University Institutional Review Board (IRB-00060350). Since samples for this study involved the use of samples obtained as part of an ongoing study where written informed consent was obtained for storage and use of samples in other studies, it qualifies for a waiver of informed consent for this study. Samples from 5 patients were used in this study. All mouse studies were reviewed and approved by the Emory Institutional Animal Care and Use Committee (DAR-2001392-020615BN), which reviews the animal care and use projects. The committee adheres to specific national and international regulations regarding the ethical treatment of animals as specified by the National Institutes of Health.

## Supporting Information

S1 Fig Identification of multiple immune cell populations by flow cytometry, and effects of imatinib on DC subsets. **(A)** Schema for isolation of myeloid and lymphoid populations (Fig. 1A). Live singlet cells from collagenase-digested spleens were lineage gated by expression of NK1.1, Thy1.2, B220 and CD19 and then divided into dendritic cells and myeloid cells by expression of CD11c and CD11b. Total DCs were subdivided as CD8^+^ DC (CD11c^hi^, Ly6C^−^, CD8^+^, CD11b^−^), CD8a^−^ DC (CD11c^hi^, Ly6C^−^, CD8^−^, CD11b^+^), and pDC (B220^+^, Ly6C^+^, CD19^−^, Thy1.2^−^, CD11c^int^, and CD11b^−^). CD11b^hi^ myeloid cells were subdivided into eosinophils (SSC^hi^, Gr-1^int^, Ly6C^int^), monocytes (SSC^lo^, Ly6C^hi^, Gr-1^int^, CD11c^−/int^) and neutrophils (SSC^int^, Ly6C^int^, Gr-1^hi^). **(B)** Representative flow cytometry contour plots gated on total live singlet splenocytes from a control animal, an animal treated with imatinib (66mg/kg/d), a Mm-infected animal, and an infected animal treated with imatinib. The box on the right of each panel represents neutrophils. The total number of cells, and the number of neutrophils is listed below each plot, and the percentage of neutrophils relative to total live cells is shown within the box **(C)** Effects of imatinib on DC cell subtypes. Beginning 24h post-treatment mice were either injected in the tail vein with 10^5^ CFU Mm 1218R or left uninfected. CD8^+^ DC (CD11c^hi^ CD8^+^), CD8^-^ DC (CD11c^hi^ CD11b^+^ CD8^−^) and pDC (CD11c^lo^ CD11b^−^ B220^+^ and Ly6C^+^) numbers were enumerated by flow cytometry in the blood (top panel) or spleen (bottom panel) at d7 post infection or treatment. Combined data from two to three independent experiments are presented with 6 mice per condition. A Mann-Whitney test was used for pairwise comparisons, and a Kruskal Wallis test for multiple comparisons.
(TIF)

S2 Fig Gating schemes for isolation of mature myeloid and lymphoid cells, myeloid precursors and progenitors in bone marrow. **(A)** Gating scheme for isolation of mature myeloid and lymphoid cells from bone marrow in Fig. 2. **(B)** DC subsets from bone marrow of imatinib-treated or infected mice. C57Bl/6 mice were administered imatinib at 66 mg/kg/d or left untreated. Beginning 24h after onset of drug, mice were either injected in the tail vein with 10^5^ CFU Mm 1218R or left uninfected. At 7 days post-treatment bone marrow was collected from femurs. CD8^+^, CD8^-^ and pDCs were enumerated by flow cytometry. The line in each data set represents the median. A Mann-Whitney test was used for pairwise comparisons, and a Kruskal-Wallis test for multiple comparisons. Combined data from two independent experiments are shown.
(TIF)

S3 Fig Gating scheme for isolation of myeloid precursors in bone marrow. **(A)** Representative flow cytometry contour plot of bone marrow subset gating based on CD45 expression and side scatter (SSC)**:** lymphocytes (yellow), monocytes (green), promyelocytes (orange), myelocytes/metamyelocytes (pink), mature neutrophils (red), and total granulocytes including all neutrophil progenitor populations and mature neutrophils (blue). **(B)** Frequency of total live bone marrow cells of subsets. Combined data from two independent experiments are presented with n = 6 mice per condition. Bar in each data set represents the median+/-SEM. A Mann-Whitney nonparametric test was used to determine significance.
(TIF)

S4 Fig Gating scheme for isolation of hematopoietic stem cells, and myeloid progenitors in bone marrow. **(A)** Gating Scheme for isolation of LSK, HSC, MPP1, MPP2, MPP3 and MPP4 cells from bone marrow (adapted from [41]). Following selection of the lineage negative population, cells were phenotyped for expression of c-Kit and Sca-1 to select the LSK cells (lineage^−^, c-Kit^+^ and Sca-1^+^). LSK cells were then divided into populations A, B and C according to their expression of CD48 and CD150. The co-expression of CD34 and CD135 were then used to delineate the HSC and MPP subpopulations (MPP1-4). **(B)** Representative plots of LSK cells with or without imatinib. Plots show selection of linage negative cells phenotyped with c-Kit and Sca-1. LSK cells, which are Sca-1^+^ and c-Kit^+^, are delineated by the boxes with the overall percentage indicated. The panels are representative, and derived from a control animal (left panel), or and animal treated with imatinib (66mg/kg/d) for 7d (right panel). **(C)** Effects of ACK2 antibody during infection with Mm. ACK2 or 2A3 (isotype control) (10mg) was injected intravenously every 48h for six days prior to infection with Mm. CFU were measured 48 hours later. Data shown are from a representative experiment. Differences in CFU between the isotype and ACK2-treated animals did not reach the 0.05 level of statistical significance using a Mann-Whitney nonparametric test.
(TIF)

S5 Fig Neutrophils are not activated by imatinib treatment. **(A)** C57Bl/6 mice were treated as in Fig. 1A. Activation status was assessed by surface expression of CD66b (secondary granules), CD63 (primary granules) and apoptosis by intracellular staining for caspases 3/7 activity of the total neutrophils (Ly6G^+^Ly6C^+^) from bone marrow (left) and blood (right). Combined data from two independent experiments are presented with 6 mice per condition. The line in each data set represents the median. A Mann-Whitney nonparametric test was used to determine significance. **(B-C)** Depletion of neutrophils using the Anti-Ly6G (clone 1A8). Anti-Ly6G (1A8), a neutrophil specific marker, or control Ig (clone 2A3) were administered to naïve or imatinib-treated animals at a dose of 300μg I.P. one day prior to administration of imatinib (66mg/kg/d) and again 2 days later. At d3 following administration of imatinib, blood was analyzed for presence of neutrophils. **(B)** Representative flow cytometry plots measuring blood neutrophil depletion following administration of 1A8 antibody to naïve or imatinib treated mice. Neutrophils were identified by anti-Ly6G (clone 1A8), anti-Gr-1 or anti-DEC-205 fluorescent-conjugated antibody staining on singlet, non-lymphocyte, non-eosinophil, non-DC and non-CD11c^+^ monocyte blood cells. **(C)** Quantitation of neutrophil numbers under conditions described in b, using anti-Ly6G(1A8) and anti-DEC-205 staining to evaluate neutrophil numbers. Data shown in b and c are from a representative experiment with four to five animals per group. A Kruskal-Wallis nonparametric test was used to determine significance.
(TIF)

S6 Fig Effect of imatinib on infection with *Franciscella spp*. *in vitro* and *in vivo*. Imatinib does not decrease bacterial load of pathogenic *Francisella spp*. *in vitro*. **(A)** Growth of LVS in liquid broth for 48h with or without 10μM imatinib. Growth was assessed as optical density at 600nm (OD_600_) at the indicated time points. **(B)** Growth of LVS in cultured J774A.1 macrophages with or without 10μM imatinib for 4 or 24 hours. **(C)** *F*. *novicida* was grown in liquid broth for 48h +/- 10μM imatinib. Growth was assessed as optical density at 600nm (OD_600_) at the indicated time points. **(D)** J774A.1 macrophages were treated with 10μM imatinib or left untreated and infected with *F*.*novicida*. At 4 and 24 hours CFU was determined. The limit of detection of the assay was 100 colonies. The line in each data set represents the median. A Mann-Whitney nonparametric test was used to determine significance. Combined data from three independent experiments are shown. **(E)** Effects of imatinib dose on LVS infection *in vivo*. C57Bl/6 mice were treated with imatinib at 200mg/kg/d or water for 7d prior to infection and for the duration of the experiment, and then injected subcutaneously with ~2x10^5^ LVS. After 5d, skin and spleens were collected and CFU/gram of tissue was determined. The limit of detection of the assay was 100 CFUs. **(F)** Effects of imatinib at 66mg/kg/d on T cells, B cells and NK cells upon infection with LVS. Data from a representative experiment are shown.
(TIF)
