# Supplementary figures and images for: Low Doses of Imatinib Induce Myelopoiesis and Enhance Host Anti-microbial Immunity
Source: PLoS Pathog. 2015 Mar 30;11(3):e1004770. doi: 10.1371/journal.ppat.1004770 (PMC4379053; doi:10.1371/journal.ppat.1004770)

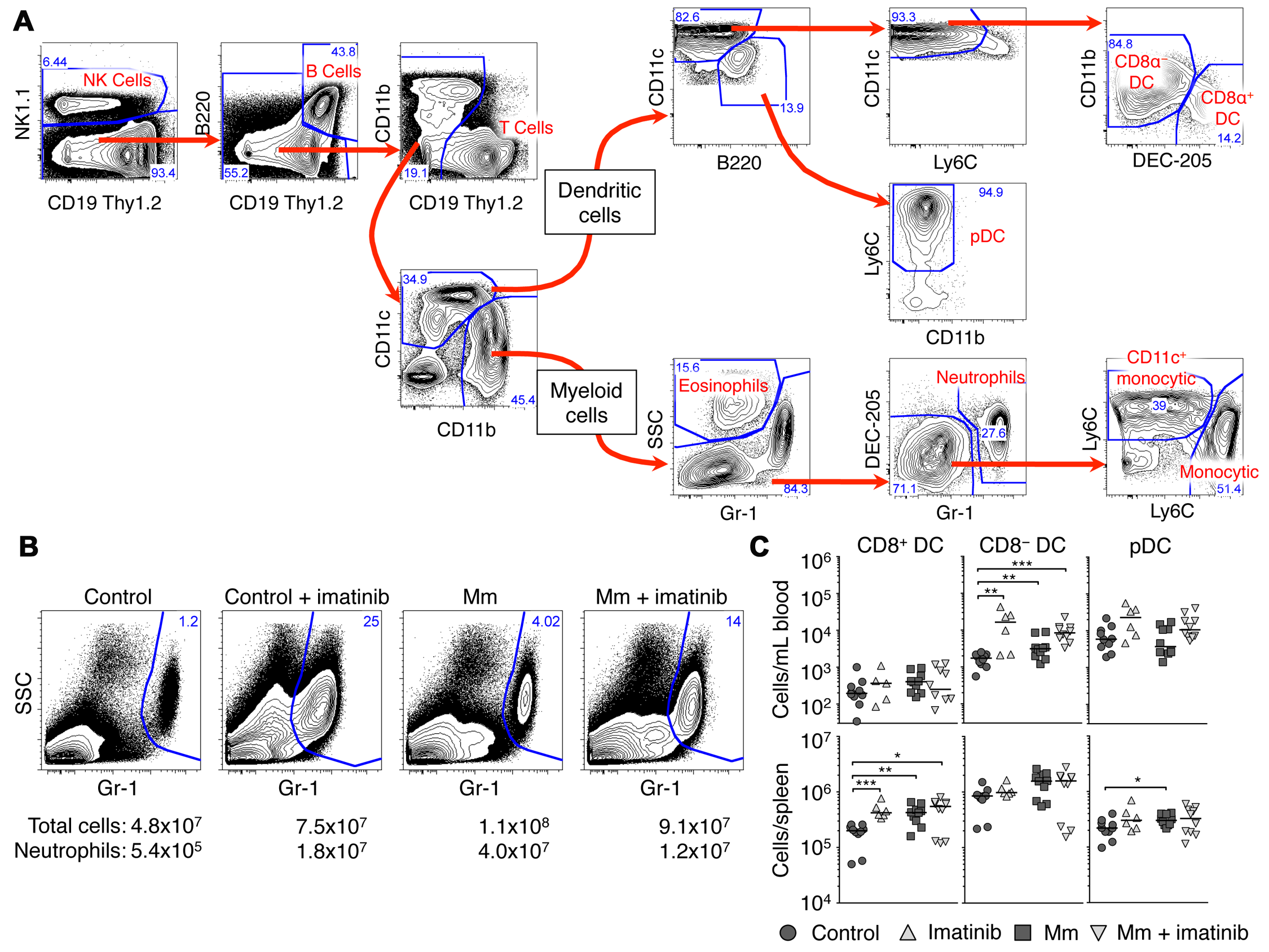

Supplement: S1 Fig — (A) Schema for isolation of myeloid and lymphoid populations (Fig. 1A). Live singlet cells from collagenase-digested spleens were lineage gated by expression of NK1.1, Thy1.2, B220 and CD19 and then divided into dendritic cells and myeloid cells by expression of CD11c and CD11b. Total DCs were subdivided as CD8+ DC (CD11chi, Ly6C−, CD8+, CD11b−), CD8a− DC (CD11chi, Ly6C−, CD8−, CD11b+), and pDC (B220+, Ly6C+, CD19−, Thy1.2−, CD11cint, and CD11b−). CD11bhi myeloid cells were subdivided into eosinophils (SSChi, Gr-1int, Ly6Cint), monocytes (SSClo, Ly6Chi, Gr-1int, CD11c−/int) and neutrophils (SSCint, Ly6Cint, Gr-1hi). (B) Representative flow cytometry contour plots gated on total live singlet splenocytes from a control animal, an animal treated with imatinib (66mg/kg/d), a Mm-infected animal, and an infected animal treated with imatinib. The box on the right of each panel represents neutrophils. The total number of cells, and the number of neutrophils is listed below each plot, and the percentage of neutrophils relative to total live cells is shown within the box (C) Effects of imatinib on DC cell subtypes. Beginning 24h post-treatment mice were either injected in the tail vein with 105 CFU Mm 1218R or left uninfected. CD8+ DC (CD11chi CD8+), CD8- DC (CD11chi CD11b+ CD8−) and pDC (CD11clo CD11b− B220+ and Ly6C+) numbers were enumerated by flow cytometry in the blood (top panel) or spleen (bottom panel) at d7 post infection or treatment. Combined data from two to three independent experiments are presented with 6 mice per condition. A Mann-Whitney test was used for pairwise comparisons, and a Kruskal Wallis test for multiple comparisons. (TIF) [file ppat.1004770.s001.tif]

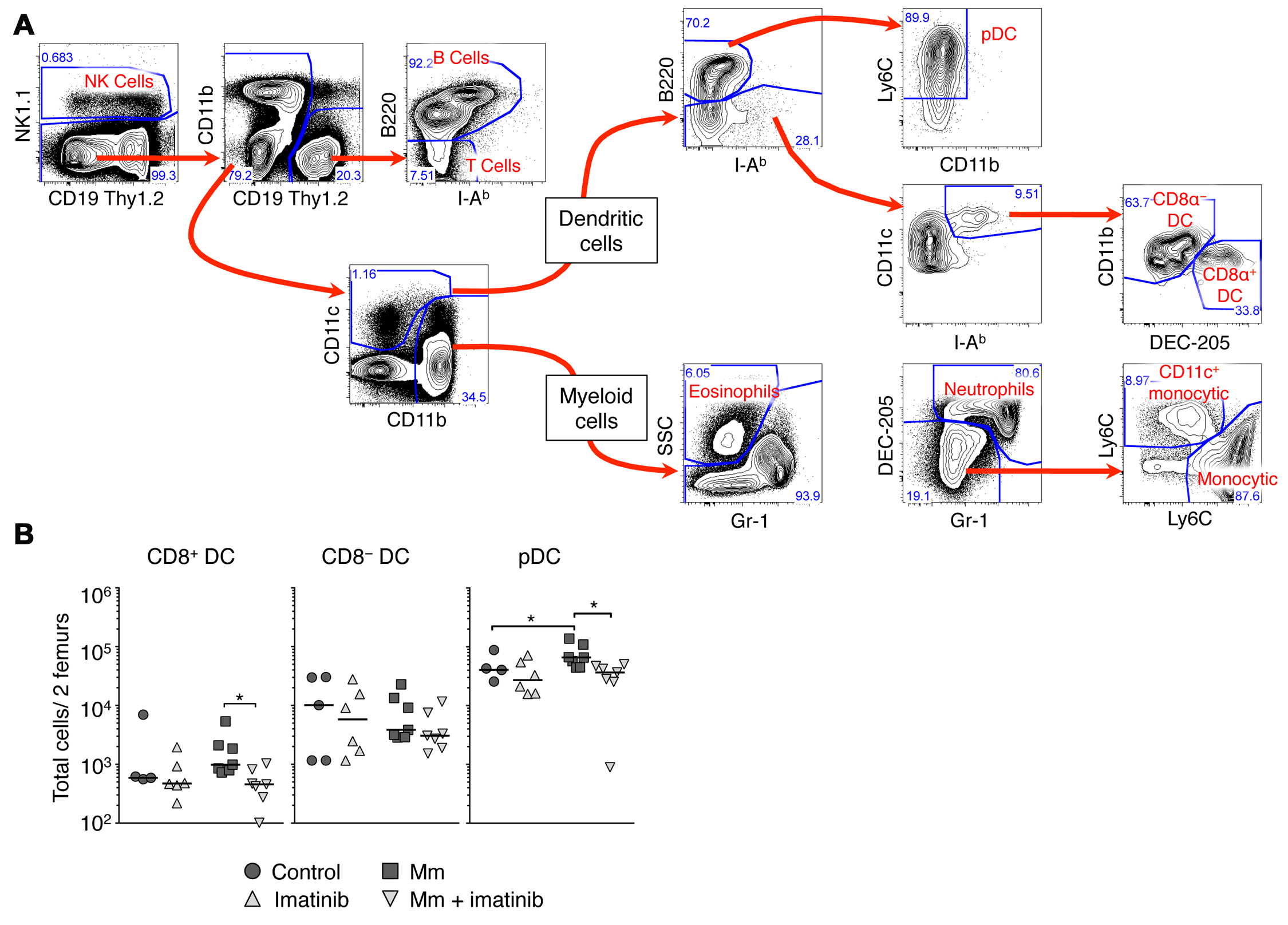

Supplement: S2 Fig — (A) Gating scheme for isolation of mature myeloid and lymphoid cells from bone marrow in Fig. 2. (B) DC subsets from bone marrow of imatinib-treated or infected mice. C57Bl/6 mice were administered imatinib at 66 mg/kg/d or left untreated. Beginning 24h after onset of drug, mice were either injected in the tail vein with 105 CFU Mm 1218R or left uninfected. At 7 days post-treatment bone marrow was collected from femurs. CD8+, CD8- and pDCs were enumerated by flow cytometry. The line in each data set represents the median. A Mann-Whitney test was used for pairwise comparisons, and a Kruskal-Wallis test for multiple comparisons. Combined data from two independent experiments are shown. (TIF) [file ppat.1004770.s002.tif]

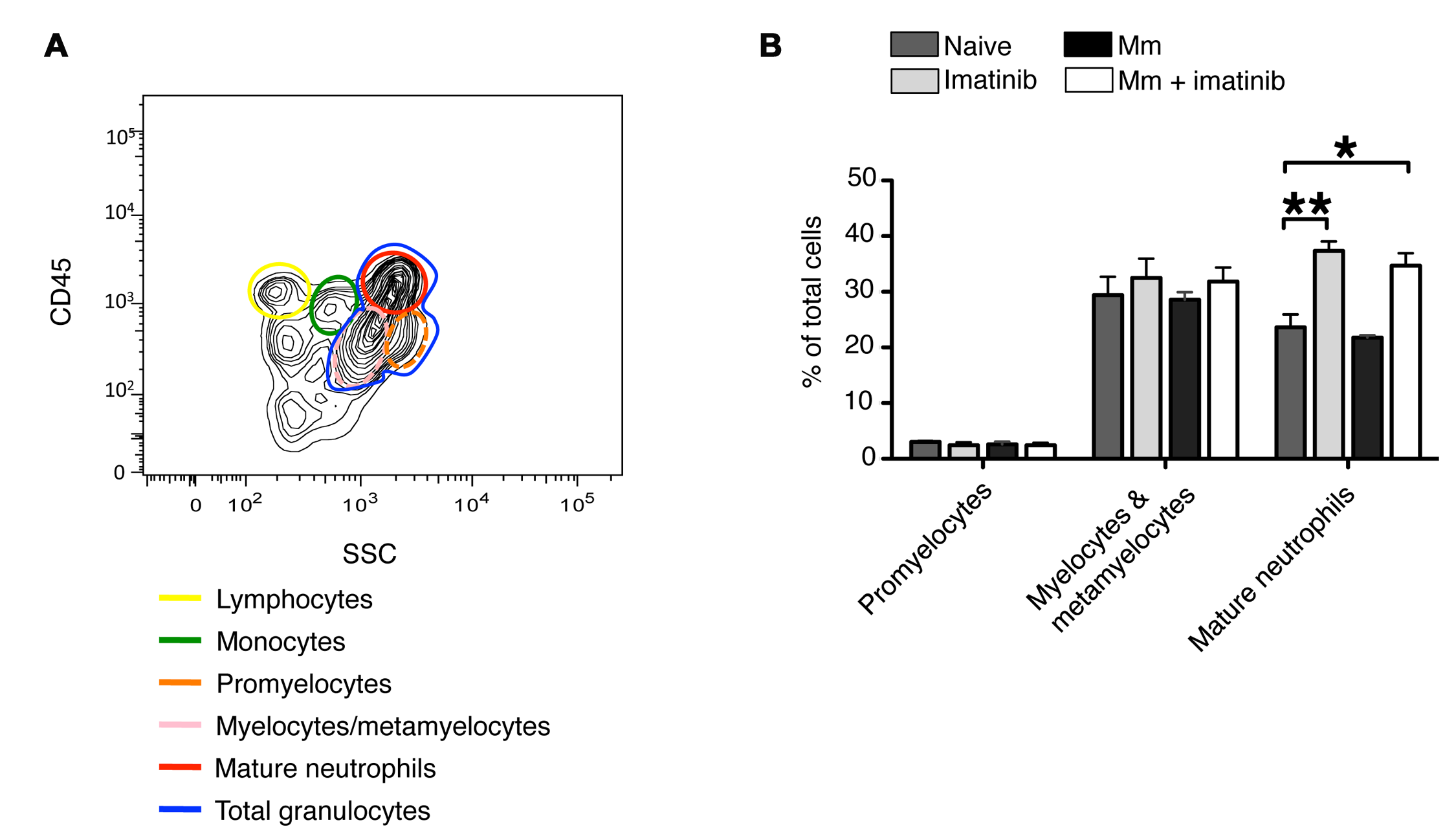

Supplement: S3 Fig — (A) Representative flow cytometry contour plot of bone marrow subset gating based on CD45 expression and side scatter (SSC): lymphocytes (yellow), monocytes (green), promyelocytes (orange), myelocytes/metamyelocytes (pink), mature neutrophils (red), and total granulocytes including all neutrophil progenitor populations and mature neutrophils (blue). (B) Frequency of total live bone marrow cells of subsets. Combined data from two independent experiments are presented with n = 6 mice per condition. Bar in each data set represents the median+/-SEM. A Mann-Whitney nonparametric test was used to determine significance. (TIF) [file ppat.1004770.s003.tif]

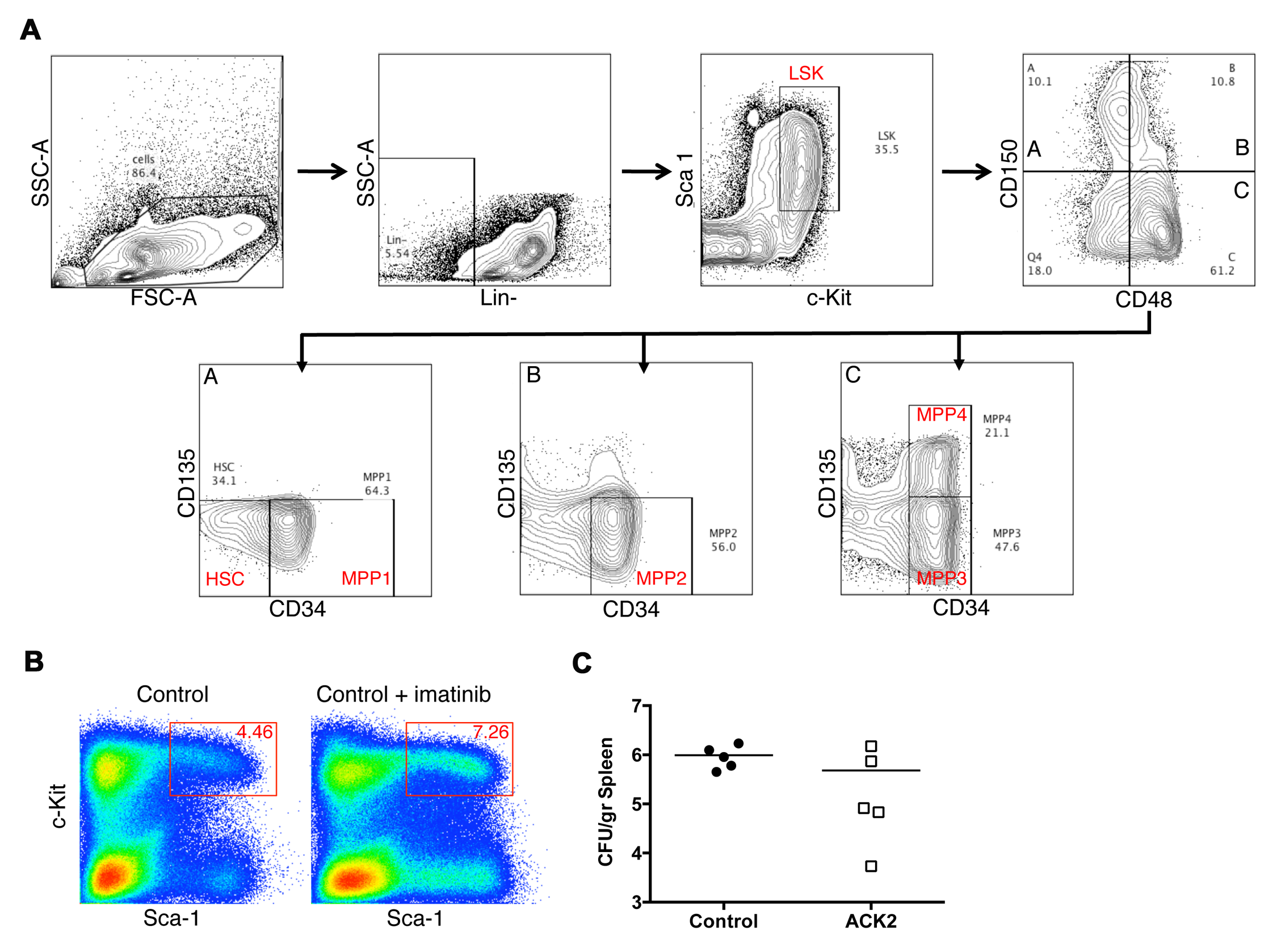

Supplement: S4 Fig — (A) Gating Scheme for isolation of LSK, HSC, MPP1, MPP2, MPP3 and MPP4 cells from bone marrow (adapted from [41]). Following selection of the lineage negative population, cells were phenotyped for expression of c-Kit and Sca-1 to select the LSK cells (lineage−, c-Kit+ and Sca-1+). LSK cells were then divided into populations A, B and C according to their expression of CD48 and CD150. The co-expression of CD34 and CD135 were then used to delineate the HSC and MPP subpopulations (MPP1-4). (B) Representative plots of LSK cells with or without imatinib. Plots show selection of linage negative cells phenotyped with c-Kit and Sca-1. LSK cells, which are Sca-1+ and c-Kit+, are delineated by the boxes with the overall percentage indicated. The panels are representative, and derived from a control animal (left panel), or and animal treated with imatinib (66mg/kg/d) for 7d (right panel). (C) Effects of ACK2 antibody during infection with Mm. ACK2 or 2A3 (isotype control) (10mg) was injected intravenously every 48h for six days prior to infection with Mm. CFU were measured 48 hours later. Data shown are from a representative experiment. Differences in CFU between the isotype and ACK2-treated animals did not reach the 0.05 level of statistical significance using a Mann-Whitney nonparametric test. (TIF) [file ppat.1004770.s004.tif]

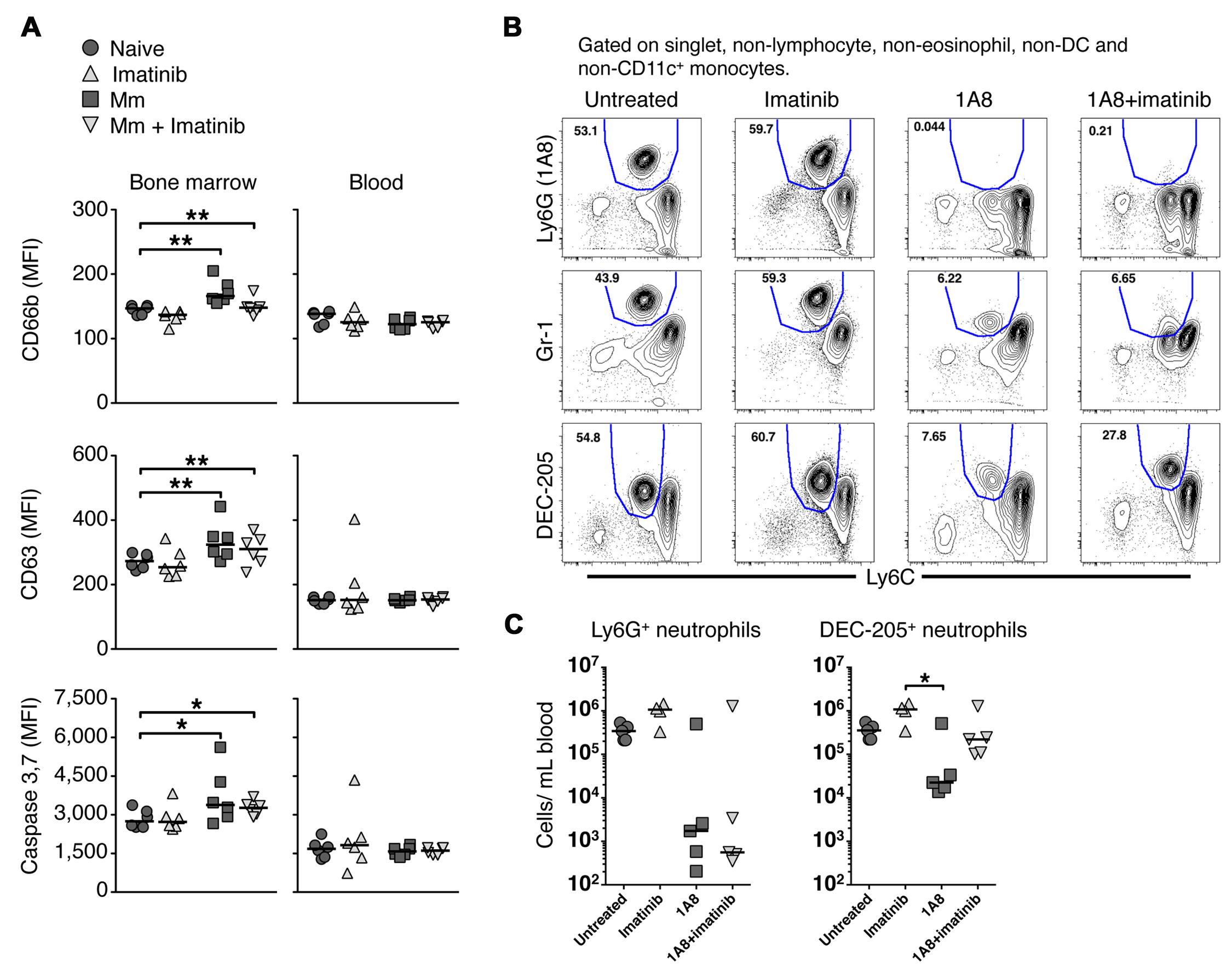

Supplement: S5 Fig — (A) C57Bl/6 mice were treated as in Fig. 1A. Activation status was assessed by surface expression of CD66b (secondary granules), CD63 (primary granules) and apoptosis by intracellular staining for caspases 3/7 activity of the total neutrophils (Ly6G+Ly6C+) from bone marrow (left) and blood (right). Combined data from two independent experiments are presented with 6 mice per condition. The line in each data set represents the median. A Mann-Whitney nonparametric test was used to determine significance. (B-C) Depletion of neutrophils using the Anti-Ly6G (clone 1A8). Anti-Ly6G (1A8), a neutrophil specific marker, or control Ig (clone 2A3) were administered to naïve or imatinib-treated animals at a dose of 300μg I.P. one day prior to administration of imatinib (66mg/kg/d) and again 2 days later. At d3 following administration of imatinib, blood was analyzed for presence of neutrophils. (B) Representative flow cytometry plots measuring blood neutrophil depletion following administration of 1A8 antibody to naïve or imatinib treated mice. Neutrophils were identified by anti-Ly6G (clone 1A8), anti-Gr-1 or anti-DEC-205 fluorescent-conjugated antibody staining on singlet, non-lymphocyte, non-eosinophil, non-DC and non-CD11c+ monocyte blood cells. (C) Quantitation of neutrophil numbers under conditions described in b, using anti-Ly6G(1A8) and anti-DEC-205 staining to evaluate neutrophil numbers. Data shown in b and c are from a representative experiment with four to five animals per group. A Kruskal-Wallis nonparametric test was used to determine significance. (TIF) [file ppat.1004770.s005.tif]

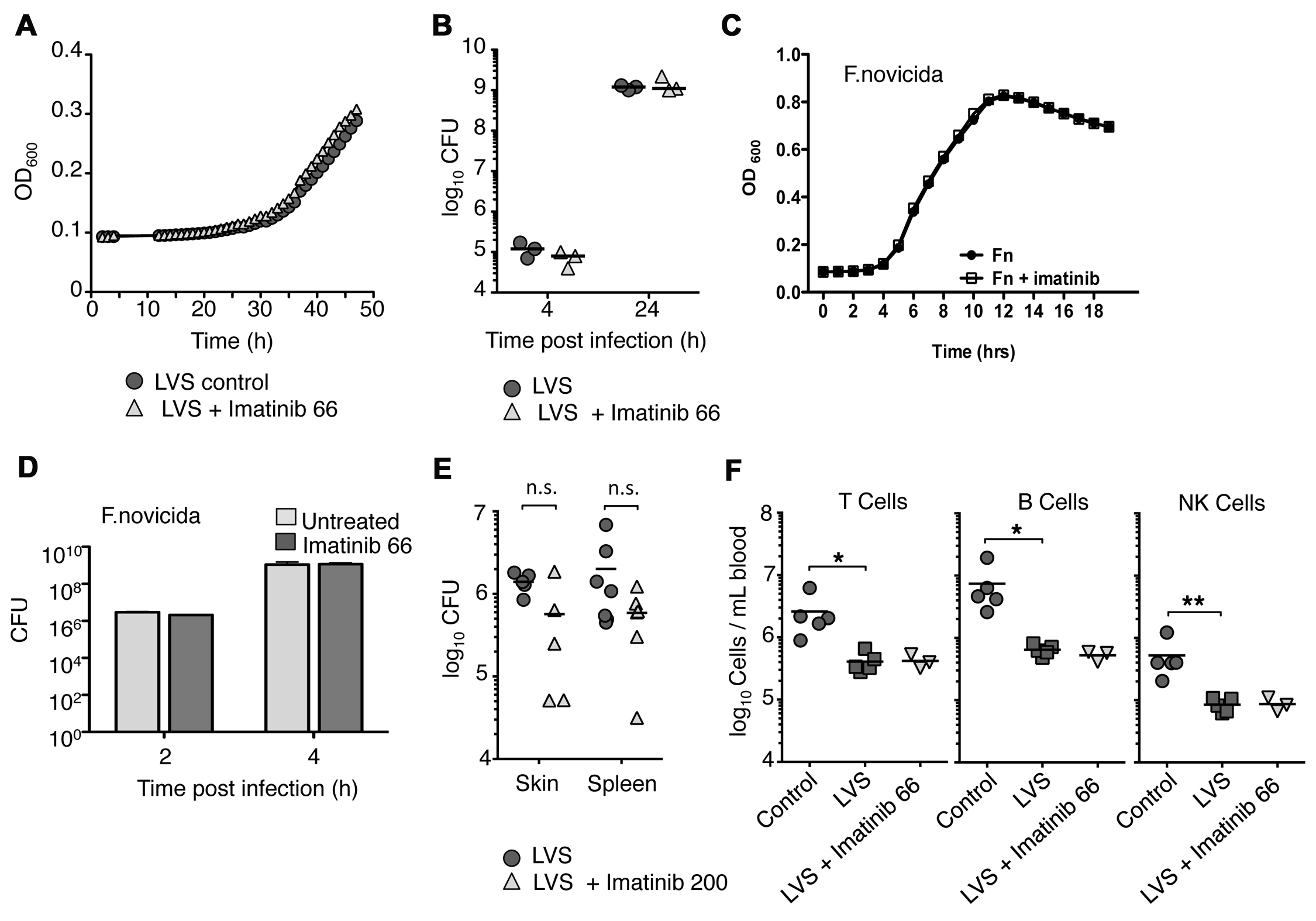

Supplement: S6 Fig — Imatinib does not decrease bacterial load of pathogenic Francisella spp. in vitro. (A) Growth of LVS in liquid broth for 48h with or without 10μM imatinib. Growth was assessed as optical density at 600nm (OD600) at the indicated time points. (B) Growth of LVS in cultured J774A.1 macrophages with or without 10μM imatinib for 4 or 24 hours. (C) F. novicida was grown in liquid broth for 48h +/- 10μM imatinib. Growth was assessed as optical density at 600nm (OD600) at the indicated time points. (D) J774A.1 macrophages were treated with 10μM imatinib or left untreated and infected with F.novicida. At 4 and 24 hours CFU was determined. The limit of detection of the assay was 100 colonies. The line in each data set represents the median. A Mann-Whitney nonparametric test was used to determine significance. Combined data from three independent experiments are shown. (E) Effects of imatinib dose on LVS infection in vivo. C57Bl/6 mice were treated with imatinib at 200mg/kg/d or water for 7d prior to infection and for the duration of the experiment, and then injected subcutaneously with ~2x10^5^ LVS. After 5d, skin and spleens were collected and CFU/gram of tissue was determined. The limit of detection of the assay was 100 CFUs. (F) Effects of imatinib at 66mg/kg/d on T cells, B cells and NK cells upon infection with LVS. Data from a representative experiment are shown. (TIF) [file ppat.1004770.s006.tif]
